# What adaptation to research is needed following crises: a comparative, qualitative study of the health workforce in Sierra Leone and Nepal

**DOI:** 10.1186/s12961-018-0285-1

**Published:** 2018-02-07

**Authors:** Joanna Raven, Sushil Baral, Haja Wurie, Sophie Witter, Mohamed Samai, Pravin Paudel, Hom Nath Subedi, Tim Martineau, Helen Elsey, Sally Theobald

**Affiliations:** 1 Department of International Health, Liverpool School of Tropical Medicine, Liverpool, United Kingdom; 2 Health Research and Social Development Forum, Kathmandu, Nepal; 3 College of Medicine and Allied Health Sciences, University of Sierra Leone, Freetown, Sierra Leone; 4 Institute of Global Health and Development, Queen Margaret University, Edinburgh, United Kingdom; 5 Leeds Institute of Health Sciences, University of Leeds, Leeds, United Kingdom

**Keywords:** Fragility, Social justice, Health workers, Crisis, Nepal, Sierra Leone, Resilience

## Abstract

**Background:**

Health workers are critical to the performance of health systems; yet, evidence about their coping strategies and support needs during and post crisis is lacking. There is very limited discussion about how research teams should respond when unexpected crises occur during on-going research. This paper critically presents the approaches and findings of two health systems research projects that explored and evaluated health worker performance and were adapted during crises, and provides lessons learnt on re-orientating research when the unexpected occurs.

**Methods:**

Health systems research was adapted post crisis to assess health workers’ experiences and coping strategies. Qualitative in-depth interviews were conducted with 14 health workers in a heavily affected earthquake district in Nepal and 25 frontline health workers in four districts in Ebola-affected Sierra Leone. All data were transcribed and analysed using the framework approach, which included developing coding frameworks for each study, applying the frameworks, developing charts and describing the themes. A second layer of analysis included analysis across the two contexts, whereas a third layer involved the research teams reflecting on the approaches used to adapt the research during these crises and what was learned as individuals and research teams.

**Results:**

In Sierra Leone, health workers were heavily stigmatised by the epidemic, leading to a breakdown of trust. Coping strategies included finding renewed purpose in continuing to serve their community, peer and family support (in some cases), and religion. In Nepal, individual determination, a sense of responsibility to the community and professional duty compelled staff to stay or return to their workplace. The research teams had trusting relationships with policy-makers and practitioners, which brought credibility and legitimacy to the change of research direction as well as the relationships to maximise the opportunity for findings to inform practice.

**Conclusions:**

In both contexts, health workers demonstrated considerable resilience in continuing to provide services despite limited support. Embedded researchers and institutions are arguably best placed to navigate emerging ethical and social justice challenges and are strategically positioned to support the co-production of knowledge and ensure research findings have impact.

**Electronic supplementary material:**

The online version of this article (10.1186/s12961-018-0285-1) contains supplementary material, which is available to authorized users.

## Background

The health workforce is critical to the delivery of healthcare and strong robust health systems. Research on health systems strengthening, including how best to retain, strengthen and motivate health workers at different levels of the health system, is increasing [1–5]. We live in an uncertain and fragile world where crises, including war, natural disasters and epidemics, with wide reaching repercussions for health systems resilience and health workers’ experiences and the ability to deliver critical services, are arguably increasing.

### Health workers in crisis

There is evidence about how health workers respond to epidemics in high-income settings; behaviour is shaped, for example, by fear of contracting disease(s), concern for family health, isolation and information on risks [6–9]. However, further research is required to understand the factors that influence health workers’ decisions to stay at the frontline and deliver care in low-income settings. Recent research on health workers’ experience of Ebola refers to feelings of sadness, need for psycho-social support, and weakened trust within and across health systems and communities [10]. There is also research on health workers in natural disasters and emergency settings [11–14]. Nevertheless, much of this literature on epidemics and natural disasters focusses on health workers’ experiences and challenges, rather than coping strategies and mechanisms to support them. Recent research has highlighted strategies that underpin health workers’ decision to stay serving during the war in northern Uganda [15] and Sierra Leone [16]. Coping strategies in both settings included notions of personal faith, which underpinned a strong sense of personal service to communities in all circumstances, and family support. Given the centrality of health workers to the success of health systems, further research about how to retain, value and support this group during and post crisis is required.

### Co-production of knowledge

The theory of co-production, first put forward in the 1970s, conceptualised as “*the process through which inputs used to produce a good or service are contributed by individuals who are not ‘in’ the same organisation*” ([17], p. 1073). In health, co-production is described as a way of working together to improve health and of creating user-led, people-centred healthcare services. In recent years, it has also been used to describe the growing engagement of policy-makers and practitioners in applied research [18]. Co-production of research can lead to evidence that responds to the needs of the users, that the users consider as more credible and that they feel confident to utilise. Co-production can generate powerful synergies, offer illuminating insights into critical contemporary issues, and bring the worlds of academia and practice closer together [19].

There are some core elements of co-production. First, the relationships that allow co-production to happen are crucial [20]. The relationships between users and producers are reciprocal and mutually beneficial; they each bring potentially unique contributions, and they recognise that they can achieve more by working together than they can apart [18, 21]. Trust is crucial to the development of these types of relationship. Second, the users of evidence are seen as active agents rather than passive recipients; they are involved in the formulation of the research question, study design and analysis [18]. Third, the composition of the team needs to demonstrate local credibility and a good knowledge of the context in order to bring about evidence-based change [22] and is linked to the concept of embeddedness.

### Research partnerships, co-production and being responsive to crisis

The importance of context-responsive approaches within applied social science research is well established [23], as it is also within implementation research, which often applies social science approaches. Indeed, WHO guidelines emphasise that it is the “*interaction between real world and the intervention being studied that sets* [implementation research] *apart from routine monitoring*” [24]. However, none of these guidelines provide insights on how to work strategically in partnerships in situations where a major disaster hits the study sites, nor the implications and responsibilities of research teams. This is an area that requires more attention to guide researchers in pragmatic and ethically informed practice and knowledge co-production.

### Evidence gaps

There are two important evidence gaps. Firstly, how do health workers cope in times of crisis, and how can they best be enabled to continue their work within different contexts and in response to different crises? Secondly, how should research teams respond when unexpected crises occur during on-going research on the health workforce and health systems strengthening?

This paper critically presents the approaches and findings of two health systems research projects that explored and evaluated health worker performance. In both contexts, crises occurred mid research (a major earthquake in Nepal in 2015 and an Ebola outbreak in Sierra Leone 2014–2015) with major implications for both health systems strengthening and health workers, as well as the ongoing research. Herein, we present the methods and their adaptation in response to crisis, our findings, and the methodological, social justice and ethical lessons learnt on conducting applied social science research and the co-production of knowledge in these contexts.

## Methods

### Sierra Leone

#### Original study

Health systems research on health workers and incentives was conducted in Sierra Leone from 2011 to 2014. This included a health worker incentive survey, analysis of routine human resource secondary data, document review, key informant interviews, and life histories with health workers in four districts (Additional file 1: Sierra Leone original study protocol). The research was carried out by national researchers from the College of Medicine and Allied Health Sciences (COMAHS), as part of the ReBUILD consortium. COMAHS is strategically positioned to partner with the Ministry of Health and decision-makers to use research to strengthen health policies and practices.

#### The crisis

The 2014 Ebola outbreak evolved in alarming ways in Sierra Leone. The virus spread to all 14 districts and the country struggled to control the escalating outbreak against an already weak health system. Efforts made in the post-conflict period to strengthen human resources suffered a major set-back from Ebola. Sierra Leone was declared Ebola free by WHO on March 17, 2016, and the outbreak had claimed 3955 lives as of December 30, 2015 [25]. Health workers were at the forefront, and therefore exposed to a higher risk of contracting the virus [26]; indeed, by May 2015, 0.06% of Sierra Leone’s population had died due to Ebola compared with 6.85% of the country’s health workers [27].

#### Adapted study

In both Sierra Leone and Nepal, following dialogue, the focus shifted post disaster to understanding health workers’ experience of crisis, their coping strategies and how these could best be strengthened and supported.

In Sierra Leone, the aim of the adapted study was to understand the challenges to a responsive and resilient health system from a health worker perspective in the face of the recent Ebola shock, and how to build resilience to such shocks in the future. The study was conducted in four districts (Western Area, Bonthe, Kenema and Koinadugu) with a varying number of Ebola cases (Additional file 2: Sierra Leone adapted study). Twenty-five in-depth interviews were conducted with a cross section of the workforce who were providing Ebola services. We interviewed national health workers, namely doctors, nurses/midwives and health assistants, who were working in Ebola treatment centres to explore their experiences of the Ebola outbreak and its effects on their work, the facilitators and challenges within the health system, coping mechanisms, and suggestions for strengthening the health system. We also interviewed health workers working in other government facilities to understand the wider effects of the Ebola outbreak beyond the specific treatment centres. Interviews with international health workers, mostly senior level health workers such as nurses and doctors or administrative heads, captured the perceptions of outsiders with operational insights on the current functioning of service delivery in the districts. As health workers who have not worked in the Sierra Leone health system, they provided a unique and important perspective on how health workers coped with responding to the outbreak, and ways to rebuild the health system post Ebola. During March and April 2015, the research team conducted all the interviews in English, face to face, using topic guides. Written informed consent was obtained. The interviews were digitally recorded after gaining permission from the participants, and transcribed verbatim. Ethical approval was obtained from the Sierra Leone Scientific and Ethics Committee and the Liverpool School of Tropical Medicine Research Ethics Committee.

### Nepal

#### Original study

The research in Nepal focused on health workers experiences of a performance-based management system (PBMS) and health systems strengthening initiative. The PBMS was implemented during 2014 and 2015 in three districts, and the mixed method process evaluation included in-depth interviews, focus group discussions, observations, analysis of routine health service data and health worker motivation surveys (Additional file 3: Nepal original study). This research was led by Nepali researchers from the Health Research and Social Development Forum Nepal, in partnership with the Ministry of Health, Liverpool School of Tropical Medicine and the University of Leeds. To strengthen partnerships and maximise embedding of the research process, the team developed several joint working forums with ministry and other key stakeholders at both national and district levels.

#### The crisis: earthquake

On April 25, 2015, a massive earthquake measuring 7.8 on the Richter scale was experienced in Nepal, with continued aftershocks. Fourteen districts were heavily affected by the earthquake, while many others were also reported to be affected. Almost 9000 people died and over 21,900 were injured [28]. Many households and health facilities were destroyed or severely damaged. Rasuwa district, one of our study sites, was heavily affected, and recorded 597 deaths and approximately 8000 injured people. All of the 20 facilities in the district were damaged [29], two health workers died, nine were injured and two remain missing [28, 30].

#### Adapted study

The research on developing PBMS with health workers was conducted in three districts. Only one of these, Rasuwa, was badly affected by the earthquake, so it was here that the study adaptations took place. Rasuwa is a remote and mountainous district, with challenges in road access and communication. We aimed to explore health workers’ experiences of implementing PBMS pre- and post-earthquake to understand working environments and any factors influencing changes to service delivery (Additional file 4: Nepal adapted study). The study was conducted in five facilities with varying degrees of damage caused by the earthquake, and included a primary healthcare centre and four health posts. Qualitative methods were used, including observations of the conditions and working practices within health facilities; six semi-structured interviews with health workers (auxiliary nurse midwives, auxiliary health workers), five with managers and three with health facility management committee members. Managers at primary healthcare centres and health posts provide a dual role of management and healthcare delivery. This sample represents the typical workforce operating in these rural areas of Nepal. The interviewees, after providing written informed consent, were asked to describe the whole experience of the earthquake, how they delivered healthcare services in the immediate post-earthquake period and coping strategies adopted by health workforce while responding to service delivery needs. The interviews were conducted in locations chosen by the participants, such as in health centres, offices or homes, they were digitally recorded following permission from each participant, and detailed notes were taken. The recordings were transcribed verbatim, and the notes were written up in an electronic form. Ethical approval for the larger study and the adapted study was obtained through the WHO ethical review committee and the Ministry of Health Nepal ethics committee.

#### Analysis

There were three levels of analysis for this paper. First, the data for the Sierra Leone and Nepal studies were analysed separately by the country research teams with support from the United Kingdom collaborators. In both settings, the teams reflected on the data as it was being collected and identified emerging themes through the framework qualitative analysis process [31], with most analysis being conducted at the end of the project. Coding frameworks for each study were developed literatively using themes emerging from the data, the topic guides and study objectives. The country research teams applied the frameworks to the transcripts, charts were developed for each theme, and these charts were used to describe the themes.

Second, the analysis across the two contexts was then conducted – the lead authors first identified the themes through review of the initial analysis and relevant literature [14, 16, 32] and then shared these with the other authors to interrogate and refine, these were then developed further with evidence from the two contexts, and then shared for further refinement. Consensus on key themes (e.g. health systems readiness; equipment and supplies, communications, numbers and skills of health workers; effects on health workers, namely injury or illness, fear and distress, stigma; coping mechanisms; support from communities, support from health systems) across contexts and disciplines was reached through iterative reflection and dialogue through e-mail and Skype, and over a period of several months. The iterative reflection involved the authors reviewing the themes, and checking that the data supports the themes. By involving all authors, who had different professional, personal and geographical backgrounds, we ensured that different interpretations and perspectives were incorporated in the analysis [33, 34].

Third, an analysis was undertaken in which the research teams reflected on approaches and key themes emerging (research adaptation and flexibility, dialogue with policy-makers and practitioners, relationships/embeddedness) from adapting the research during these crises and what was learned as individuals and research teams [35].

In the results section, the findings are presented in two sections, the first including the key themes that emerged from the analysis across the two contexts, namely health systems readiness and impact of the crisis on health workers, and health worker coping strategies. The second section focuses on the reflection of the research teams on adapting the research during the crises, and the implications for ethics and the co-production of knowledge in crisis.

## Results

Table 1 illustrates the studies’ samples; the Nepal sample included 14 participants and the Sierra Leone sample included 25. There were more female than male participants in both studies, reflecting the composition of the workforce at this level of the health system.

**Table 1:** Participants of the studies in Sierra Leone and Nepal

Sierra Leone				Nepal			
National from Ebola treatment centres	National from other health facilities	International	Total	Health workers	Managers/health workers	Members of the Health Facility Management Committee	Total
11 (7 female; 4 male)	11 (7 female; 4 male)	3 (1 female; 2 male)	25 (15 female; 10 male)	6 (5 female; 1 male)	5 (2 female; 3 male)	3 (1 female; 2 male)	14 (8 female; 6 male)

### Findings from the Sierra Leone and Nepal studies

#### Health systems readiness and impact of the crisis on health workers

In Sierra Leone, specific challenges related to readiness of the system to manage the Ebola outbreak were reported. These included a lack of triage facilities, isolation and treatment beds, training in infection prevention and control, and protective equipment as well as limited numbers of laboratories, instruments and supplies. Further, the lack of knowledge and misconceptions about Ebola also contributed to health workers’ fear of the disease and how to protect themselves from infection as well as anxiety when caring for patients.

“*I've never seen an Ebola patient before in my whole life and it happened last year, July… I was on call that day, I had to wear Personal Protective Equipment after Googling it for 30 minutes …read how to put it on and how to remove it.*” (Health Worker, Sierra Leone, Male)

“*First, it was fear, fear of everything, not knowing much about this type of disease that have broken out in the country… but when we started learning about the Ebola it became a little better, but it was difficult.*” (Health Worker, Sierra Leone, Female)

Respondents reported several negative effects on health workers. Many community members believed that Ebola was spread by health workers through contact, exchanging blood or injections, and were frightened of health workers dressed in protective gear. Health workers felt stigmatised, isolated and ostracised, for example, by not being allowed to use the village well for their water, being asked to leave their rented accommodation, and not being allowed to use taxis. Other effects included isolation from families to protect them from infection, relatives discouraging health workers from working, the trauma of watching colleagues die and fearing for yourself, and economic hardship due to reduced earnings. In the workplace, health workers often reported stress and overload, and a continued struggle to get the supplies they needed, and some reported distrust between staff, for example, between those in general versus treatment facilities.

“*… colleagues in the general ward they were really intimidating us. If I walked through this corridor, they will just move and just give a space for me to pass… It is because of the Ebola so they are all afraid. We are talking to them that we are not carrying the virus with us, they need to courage us, they need to talk to us, we are fighting for them.*” (Health Worker, Sierra Leone, Male)

On a positive note, some respondents reported improved skills and knowledge in triage, management of Ebola, and infection prevention and control measures through the training workshops and clinical practice.

In Nepal, post-earthquake, many health teams were able to continue to provide healthcare services from temporary shelters in open spaces, tents or less damaged buildings within the facility compound. Health workers reported that they continued providing services because they could see that people were suffering, and they felt it was their duty as health workers to help them. However, health workers and managers faced many challenges, including a lack of safe accommodation as the buildings were damaged, food shortages, and limited or damaged supplies and drugs. The earthquake and subsequent landslides blocked paths and roads, making it impossible to refer seriously injured patients until the roads were cleared, sometimes up to a week later. Communications with the district health office were hindered by the disrupted mobile network.

“*On the day of the earthquake we three health workers were in the facility, and around 3 o'clock* [Earthquake struck at 11:56] *we started providing services to the patients. Minor cases were managed and also we did what we could do for the major cases but could not refer them to higher centre for a week because we had no other option.*” (Health worker, Health post, Nepal, Female)

#### Health worker coping strategies

In Sierra Leone, several strategies that helped health workers cope with working during the Ebola outbreak were identified, including training, which helped health workers overcome fear and become more confident about providing care; being given the appropriate equipment to be able to do their job safely; peer, family and community support, and a social media platform (e.g. creating a WhatsApp group which was used as a platform to share supportive and encouraging messages with each other), which helped health workers deal with a range of challenges. In addition, workshops that provided emotional support and ways to deal with the social stigma associated with being a health worker; religious beliefs, including praying together before starting work, helped health workers cope with seeing patients and colleagues dying from Ebola. Finally, a risk allowance (which ranged from 500,000 Leones (approximately $70) per week for doctors, nurses, midwives, community health officers working in treatment centres and community care centres and all members of the burial team, to 100,000 Leones (approximately $13) for contact tracers) motivated some staff to work in the facilities and provided an additional income source, which helped cope with the increased cost of living. Training and collegial support emerged as key to support and coping strategies.

“*If I make a simple mistake, just a simple one, I will die. So what we have been doing is to constantly keep talking to our colleagues. You will now tell your colleague please be careful, we send text messages around, to wash our hands.*” (Health Worker, Western Area, Sierra Leone, Female)

“*The training and a lot of protective gears helped the situation. When you had a lot of protective gear you felt more confident to go in there to your patients.*” (Health Worker, Bonthe, Sierra Leone, Female)

In Nepal, health workers reported several coping strategies that enabled them to provide services immediately following the earthquake, including going together to retrieve drugs and materials from the rubble of damaged facilities and providing immediate care and treatment with limited supplies. Individual determination, a sense of responsibility to the community and professional duty compelled staff to stay or return to their workplace.

“*My own house was damaged in earthquake but I didn’t go home. As a nurse I strongly felt that I should serve people in this locality* [health facility] *at the time of such crisis. If I don’t do so I thought it would be against my professional practice and ethics.*” (Nurse, Health Post, Nepal, Female)

“*We contacted with the health facility management committee members, they helped to convince the injured and their families, and urged the community to get the first aid materials out of the rubble. Thus it was the community as well as committee that helped in resuming services.*” (Nurse, Health Post, Nepal, Female)

Health workers in Nepal received support from the district health office and external organisations, such as WHO and UNICEF, in the form of tents and medicines, but this was often delayed by at least 1 week. Most facilities received support from the community and the health facility management committee who assisted with moving all healthcare materials, drugs and equipment to the tents, and carried referred patients to the road or other health facility.

“*Almost after a week we started getting support from the district team and supporting partners in setting-up tents, medicines and other logistics.*” (Nurse, Health Post, Nepal Female)

Health managers and workers in Nepal identified several ways that could have better supported them to provide services, including providing training for members of the rapid response teams, recognising and showing gratitude for health staff’s efforts, relief agencies providing support to the health workers as well as the villagers, and supporting families where health workers or health volunteers have died or been injured.

“*Tackling disaster of this kind we should have adequate measures and preparations such as sufficient medicines, adequate staff in the health facility, ambulance, appropriate training and support mechanisms to motivate health workers*.” (Health worker, Health post, Nepal, Female)

“*Other organisations* [supporting agencies] *distribute different types of relief materials to the affected villagers but no one provides anything for health workers. Government should do something for those health workers and community health volunteers who are dead and injured. Rapid response team should have more people and training should be provided for the management of disasters.*” (Manager, Health post, Nepal, Male).

### Reflections of the research teams on adapting the research during the crises

In Nepal, following the earthquake, the focus moved from evaluating the PBMS to health workers’ motivation and readiness to deliver basic health services in the earthquake-affected Rasuwa district, as this was seen as more relevant and could feed into concurrent policy and practice discussions and decision-making. The research provided timely evidence about health workers’ experiences in responding to the crisis to policy-makers as well as to the media. In Sierra Leone, policy-makers, practitioners and international organisations supporting the response, expressed the need to understand how the state of the health system prior to the Ebola outbreak shaped experiences, and the current challenges from a health workers’ perspective. There was a need to understand factors that supported or hindered health workers’ abilities to cope with the crisis and to generate findings that could feed into the on-going crisis and support longer term rebuilding efforts. We adapted our research process accordingly, engaging policy-makers and stakeholders throughout the research process to support the co-production of knowledge and national ownership of the findings and effective research uptake. In both cases, the research was led by researchers and research institutions that were embedded in policy and practice, and who enjoyed trusting relationships with policy-makers and practitioners, which brought credibility and legitimacy to the change of research direction as well as the relationships to maximise the opportunity for findings to inform practice.

## Discussion

### Health worker responses to the crises

The natures of the crises were clearly different in Nepal (natural disaster) and Sierra Leone (epidemic); yet, joint analysis demonstrates similarities in health workers’ responses and coping strategies as well as the broader health systems response. Health workers in both contexts demonstrated admirable everyday resilience in continuing as best as they could in incredibly challenging circumstances despite very limited support, infrastructure and recognition, particularly at the local district level.

A key difference in the two contexts lies in the ways in which stigmatisation was experienced by health workers and the implications for trust and relationships with communities and fellow health workers. In Nepal, the major earthquake was followed by aftershocks and wide-reaching destruction. It was a natural disaster, affecting everybody in certain districts and arguably triggered a sense of cohesion and community action – people banded together to support each other and health workers were central to this endeavour, although they had conflicting priorities as many had families in other districts. In Sierra Leone, the highly infectious nature of Ebola bred mistrust and fear, with health workers often being at the centre of this, being particularly vulnerable to infection and highly stigmatised. Rumours were wide spread, leading to suspicion, and in many cases to a breakdown of trust between groups of health workers as well as between health workers and communities. These findings resonate with previous studies [10, 36].

### Implications for research

Fragility and the unexpected can happen at any time. We therefore need to share learning and best practice from health systems research in these settings. Critically, health systems research needs to be flexible enough to understand the context facing health workers following disasters and to facilitate a response to improve the health worker situation. Here, we discuss two overlapping areas that have emerged from our experiences of performing research in emergency contexts.

#### A responsive and flexible approach

To the research was needed, which allowed issues of importance at that time and in that context to be explored, rather than adherence to existing methods and tools. Laying a groundwork that allows for dialogue, such as building trusting relationships with policy-makers, healthcare managers and providers, and understanding the context and, in particular, being part of the context, is vital for ensuring responsive and embedded research and facilitating the co-production of knowledge in all contexts. However, within situations of crisis, flexible responses and embedded and trusting relationships are particularly critical to enable ethical reflection and knowledge co-production in ways that support health systems responses. The immediacy of knowledge sharing within these embedded approaches was particularly important in these situations of crisis as findings from the research fed quickly into Ministry of Health responses. While attempts to reduce the gap between knowledge and practice is key within concepts of co-production and action learning [37], the ‘time’ component of this gap is particularly important within crisis situations where rapid responses are needed to ensure that health systems can still function effectively.

#### Ethics, positionality and social justice

Health workers are not typically categorised as vulnerable research participants [38], but in both cases herein, they had undergone traumatic experiences, sometimes retelling these for the first time. This has the potential to be both very sensitive as well as therapeutic if done in a supportive and sensitive manner. Conducting research during a crisis raises ethical dilemmas. Health workers’ main priority is to provide healthcare services and support in the aftermath of crises. It is critical that research does not interfere with service provision, especially during crises; as shown in the results, health workers were particularly stretched within these periods. In both studies, the crisis was abating, providing some space for health workers and managers to participate in the study, although there were a few instances in Sierra Leone when the interviews were interrupted as the respondents were needed elsewhere. There is a lack of evidence to inform decisions on how best to respond to a crisis such as the Ebola outbreak or the Nepal earthquake. Research can offer reassurance about decisions made, and can challenge or provide other options to decision-makers. Such research should be designed to ensure that, wherever possible, it can be fed into decision-making processes at that time.

Positionality describes an individual’s world-view and the position they have chosen to adopt in relation to a specific research task [39]. Some aspects of positionality are culturally ascribed or fixed, for example, gender, race or nationality, whilst others such as personal life history and experiences are contextual. Researcher positionality and the interactions developed between researchers and participants shape the trustworthiness of the research endeavour in all contexts, but are particularly critical during and post crisis. The Rasuwa interviews were conducted by Nepalese researchers who had built up long-term relationships with the health workers, and had also experienced the earthquake. Again, in Sierra Leone, national researchers had built up relationships with health workers through time, and were both also engaged in the Ebola response (one as a doctor and one as a health systems researcher involved in policy dialogue and infection prevention) and in training front line health workers during the outbreak. This embedded positionality enabled the researchers and research teams to feed findings directly to key stakeholders in positions of power. For example, Sierra Leonean researchers fed findings directly into the health systems reconstruction agenda and those from Nepal advocated for stronger support for health workers, including leave of absence, and the *Nepali Health Journal*’s recognition of health workers for their work during the earthquake and its aftermath [40].

### Lessons for policy-makers and health service managers

Our findings highlight the importance of long-term psychosocial support for health workers who are responding to crisis within the workplace and the community [36]. Non-financial and professional support approaches are important during emergencies [10]; our findings highlight how valuing health workers in terms of training and networks for support within the workplace will have positive dividends. Our learning underlines the important roles that district and central health authorities play in monitoring and responding to the needs of health workers in times of crisis. For example, transferring health workers to their home areas so that they could have time with their own families who were also suffering from the destruction, or ensuring health workers from the worst affected areas have sufficient leave to recover mentally, can help reduce burn-out amongst health workers. Presenting awards to or publicly acknowledging health workers can be a positive way forward to recognise dedication and increase motivation. The findings also emphasise the need for effective leadership during crises; understanding the context, making decisions sometimes quickly, and coordinating activities across different actors including NGOs and community structures is critical to ensure that health workers are supported to continue to provide services [41].

### Challenges to embedded and responsive research

There is the potential ethical issue of the research detracting from the operational response to the crisis. Research undertaken during a crisis should provide the evidence needed for the response, and answer key questions that governments, NGOs or humanitarian agencies are asking. The research findings can then be fed to key stakeholders to shape the response in a timely manner. This requires strong relationships between researchers and policy-makers’ and practitioners; indeed, embedded research institutions who often have such relationships are strategically placed in this regard. Ensuring scientific rigour whilst being embedded in the crisis can also be challenging, as can negotiating for appropriate and timely ethical approvals for new (or amended) research protocols.

**Fig. 1:**
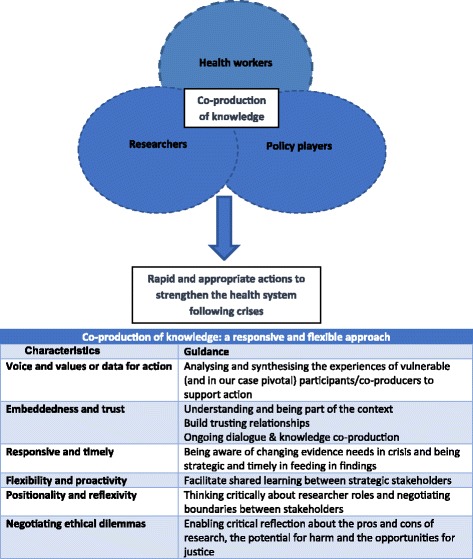
Co-production of knowledge to support health workers in fragile health systems

Figure 1 provides guidance, based on the lessons from this study, on how to follow a responsive and flexible approach to co-producing knowledge with stakeholders and, in this case, with researchers, policy-makers and health workers.

### Limitations to this research

There are several limitations of this study. We were aware that health workers were being asked to relive difficult experiences. For some health workers, this was the first opportunity to process these experiences, which proved distressing. The study was conducted as the crises were abating and we were conscious of not detracting from essential work by health workers and managers. Interviews were sometimes interrupted and cut short as respondents were needed elsewhere. This research draws on qualitative methods and explores the issues from the health workers’ and managers’ perspectives, which means that it cannot reveal other perspectives such as those of the community and patients. Co-production of knowledge usually includes engagement with a wide range of stakeholders, including communities; this was lacking in both of our case studies as we were focusing on health workers, but should be explored further, i.e. by assessing how to effectively engage community members in the co-production process.

## Conclusions

Environmental shocks are an increasing risk in many parts of the world, for which local health and research systems need to be prepared. Putting the human into human resources means valuing health workers; the research and assessment of the evidence for supporting health workers is a central part of this endeavour. We need to understand health workers’ realities and experiences, and develop pragmatic context-embedded approaches to enhance their resilience in the face of both everyday challenges and larger shocks, be they natural hazards, epidemics or conflict. As researchers, like health workers, we work in fluid and often unpredictable contexts and we need to ensure that our approaches, research partnerships and methods are fit for purpose, so we can be responsive and ensure research processes support health systems strengthening. Embedded researchers, research partnerships and knowledge co-production are central to ensuring research is both responsive and impactful in changing circumstances. There is a need for further discussion and guidance on what to do when disasters strike study sites.

## Additional files

Additional file 1: Sierra Leone original study protocol. (DOCX 215 kb)

Additional file 2: Sierra Leone adapted study protocol. (DOCX 59 kb)

Additional file 3: Nepal original study protocol. (DOCX 209 kb)

Additional file 4: Nepal adapted study protocol. (DOCX 43 kb)

## Abbreviations

COMAHS: College of Medicine and Allied Health Sciences
PBMS: Performance Based Management System
ReBUILD: Research for stronger health systems during and after conflict
