# Supplementary material for: What adaptation to research is needed following crises: a comparative, qualitative study of the health workforce in Sierra Leone and Nepal
Source: Health Res Policy Syst. 2018 Feb 7;16:6. doi: 10.1186/s12961-018-0285-1 (PMC5804047; doi:10.1186/s12961-018-0285-1)
Supplement: Supplementary file 1 — Sierra Leone original study protocol. (DOCX 215 kb) [file 12961_2018_285_MOESM1_ESM.docx]

**Additional File 1: Sierra Leone original study protocol: Understanding health worker incentives in post conflict settings**

# Background

## Rationale for study

Health worker attraction, retention, distribution and performance are arguably the most critical factors affecting the performance of a health system. In post-conflict settings, where health systems and health worker livelihoods have been disrupted, the challenges facing the establishment of the right incentive environment are particularly important, and the contextual dynamics around them especially important to understand and incorporate sensitively into policy measures. REBUILD has therefore chosen this topic as one of its focus areas for the first stage of research.

The proposal builds on insights from international literature and analysis in-country. It starts with an explanation of key terms, and based on a review of the general literature develops a conceptual framework for the research, and key research questions across the focal countries. It then summarises the research methods being developed to answer these questions in four countries: Cambodia, Sierra Leone, Uganda and Zimbabwe.

## Definition of key terms

Health worker **incentives** are variously defined, but can be broadly described as mechanisms which aim to achieve a specific change in behaviour.^1^ This can be elaborated to include “all the rewards and punishments that providers face as a consequence of the organisations in which they work, the institutions under which they operate, and the specific interventions they provide”,^2^ and, the “available means applied with the intention to influence the willingness of physicians and nurses to exert and maintain an effort towards attaining organisational goals”.^3^ The ensuing willingness is *motivation,* which develops as a result of the interaction between individual, organisational and cultural determinants.^3,4^

Health workers are generally guided by their professional conscience and professional ethos or ‘intrinsic motivation’ to do their jobs well. However, extrinsic motivation may be an important factor, particularly in certain contexts, such as where pay is low.^4,5^ Incentives are often grouped into financial or non-financial.

**Financial Incentives** are sums of money given to a worker, the amount and type or feature of which varies.^6^ Direct financial incentives include salary, pension, allowances for housing, transport and travel, child care, clothing and medical needs.^7.8^ When directly related to performance, a financial incentive (for example “pay for performance”) is conditional on taking a measurable action or achieving a predetermined performance target.^9^ Indirect financial benefits include subsidised meals, clothing, transport, childcare facilities and support for continuing education.^8^

**Non-financial incentives** are those which do not involve direct transfers with monetary value or equivalent to an individual or group.^1^ They include, career development (specialism or promotion); continuing education; hospital infrastructure (working environment); resource availability (equipment and supplies); management and supervision (positive working relationship); recognition or appreciation (at work or in the community); job security and safety.^4,7^

In this proposal we have focussed on incentives in relation to their impact on four main HR domains: the attraction, retention, distribution and performance of health workers.

# The international literature and research gaps

# Research methods

A literature review was conducted on human resources for health, focussing on incentives and post-conflict environments. A wide range of academic bibliographic databases were searched using EBSCO Discovery and PubMed in May-August 2011. Online resources for international organisations were searched for grey literature and relevant references in sourced literature were checked. The initial search term was incentives, and was used in conjunction with HRH, HRM, health workers, attraction retention, attrition, performance, motivation, and financial. The post-conflict literature was identified using the same technique in conjunction with the terms post-conflict, and fragile-state*.*

## Overview of findings

Human resources development is an important part of ReBUILDing the health sector post-conflict but has received relatively little attention in the literature and may be overlooked by decision-makers and donors.^10,11,12^ There are limited sources of literature specifically analysing health worker incentives in post-conflict states, although they are visible as one of the many elements in a wider literature of experiences from ReBUILDing health systems. Understanding the importance of human resources and the complexity of the context within which reconstruction takes place in achieving health sector redevelopment is crucial.^13^ Key issues on post-conflict states that were identified in the search on incentives are summarised briefly here as follows:

- Dealing with the imbalances which may have resulted from conflict, as well as the legacy of displacement, burn-out, attack, physical and professional isolation and outward migration of health workers.^13-19^
- The need to ReBUILD both infrastructure but also human resources management and supervision structures and the bureaucratic ability to develop and implement policy.^10,20^
- Changes to pay and control during conflict is likely to have encouraged diversification of income sources for health workers,^15^ creating challenged for public sector HR management
- Post-conflict redevelopment can exacerbate problems inherited from the pre-conflict era and the long-term effects of conflict on health and health services,^21,22^ or, alternatively, can seize the window of opportunity to address systemic issues.^23^
- The role of donors can be critical in the post-conflict period in supporting the development of weak public administrative structures or in undermining them through conflicting, multiple and/or parallel processes.^18^
- Distortion of health worker supply and salaries by the aid industry is a risk with local and foreign staff. Foreign staff can fill severe gaps in the local workforce (usually at senior level), however complaints about skills, appropriateness and capacity of expatriate health workers are commonplace, as is resentment against their higher salaries, powerful positions and decision-making freedom.^10^
- Willingness to take into account and adapt to local contextual factors (flexibility), awareness of existing (pre-conflict) distortions in health system and high level of motivation on the part of key stakeholders is important.^16,23^
- Efforts to replace lost health workers can lead to unplanned overproduction of poorly trained professionals; and educational standards may decline as emergency training inevitably produces a lower academic standard.^13^ Training may also absorb resources, enjoy support among personnel (as an important source of income), but have a negligible impact on performance.^10^
- Accurate figures and information systems are important to confirm or negate assumptions about the workforce and provide data on the skills mix and distribution of the workforce (including demographic and personal data – such as age) that can in turn help plan appropriate human resource management systems and incentives.^13,24,25^ Some cadres (midwives, or sometimes doctors) tend to be under-represented, while others are over-represented (support staff) or over-produced.
- The workforce from warring or different political factions may have established their own health services in the conflict era staffed by politically-affiliated or forcibly-recruited workers. Once the conflict is over, these personnel may need to be merged into the formal health sector.^10^  Integrating segregated workers is challenging and will require considerable preparation and sensitivity.
- Health workers without formal professional qualifications may become more common in the post-conflict setting, but they are likely to have different motivations (i.e. social respect) and respond to different incentives. The lack of formal contract can induce volunteer HWs to find alternative ways to earn a living. However, supervision is often erratic, and attrition may be high. ^10,16,24,26^
- Poor productivity as a result of absenteeism, inappropriate recruitment, poor supervision, deteriorating skills and poor regulation or low salaries is common post-conflict.^10,18,25^
- Incentives that are effective in stable health sectors can weaken and become perverse.^23^ Increased salaries are not usually sufficient to raise productivity; particularly in situations where absenteeism, poor performance, and deficient controls have prevailed during a long time, good managerial practice will also be required.
- In the post-conflict environment there are likely to be fewer opportunities for professional development and continuing education of healthcare workers, who may be discouraged due to meagre salaries, difficult circumstances, and large workloads.^14^
- Incentive strategies are likely to include increasing and standardizing salaries to attract workers and prevent outflow to the private sector; mobilizing donor funds to improve management capacity and fund incentive packages (top-up salaries and ability to gain qualifications on completion of fixed terms of office) in order to retain staff in hard to reach areas; reopening training institutions and providing scholarships to increase the pool of available workers. As in other settings, bundles of incentives which are complementary and consider living and working conditions, environments and development opportunities are likely to be more effective than individual interventions.^16,27­­-30^
- There is a growing literature on performance-based funding (PBF), including in post-conflict settings. To date the evidence on the impact of PBF remains weak,^5^ however, one multi-country study of PBF concluded that the schemes had been more successful in post-conflict than in stable settings.^31^
- Similarly, there is a growing literature on and use of performance-based contracting in a number of post conflict settings (such as Cambodia, Haiti, Afghanistan, and Rwanda). Some positive results in relation to utilization and health provider performance are noted,^31^ though the effects on health workers are rarely the focus of study.

# Research gaps and demand for research

No study to date has focused on how the decisions made, or not made, in the post-conflict period can affect the longer term pattern of attraction, retention, distribution and performance of health workers, and thus ultimately the performance of the sector. This is a gap which this proposal aims to fill. It builds on the wider ReBUILD themes and the strengths of the consortium, in that it requires a mixed methods approach, incorporating a historical perspective and sensitivity to complexity and context.

During the country situation analyses, the issues addressed by this proposal were accorded high priority by country stakeholders.

- In **Zimbabwe**, the issue of how to retain and motivate health workers (especially doctors, specialists, and midwives) is top of the agenda for stakeholders such as the Ministry of Health and Child Welfare and the Health Services Board, given on the ongoing high outward and internal migration. Understanding the different factors affecting staff in the public, municipal, rural, mission and private-for-profit sectors will be a first step to designing sustainable incentive packages.
- In **Cambodia**, rural areas and specific cadres (e.g. midwives) still present a challenge, and national strategies (e.g. the Health Strategy Plan 2008-20) highlight the need for more progress in relation to retention and performance in those areas. In a very complex policy environment, with many policies introduced nationally or in sub-regions (sometimes by donors) over the past two decades, the challenge is to gain a higher level understanding of the drivers of policy, the effects of the multiple schemes, and mechanisms underlying those effects. This will help to inform and integrate future reforms.
- In **Sierra Leone**, health worker attraction, retention, distribution and performance are also high-profile concerns, given the post-conflict legacy of shortages of workers and also low and uncontrolled remuneration. This has been addressed to some extent recently through pay uplift (2010) and through performance-based pay innovations (2011), but understanding their impact and sustainability is still required.
- In **northern Uganda**, new investments affecting health workers are proliferating, and there is considerable interest to map what is happening and to understand their effects and how they can best be managed to avoid fragmentation and distortion.

## Research aim and objectives

### Aim:

- To understand the evolution of incentives for health workers post-conflict and their effects
- To derive recommendations for different contexts on incentive environments, which will support health workers to provide access to rational and equitable health services.

### Cross-cutting research objectives:

The research questions are tailored in each country to the needs, research gaps, opportunities and contextual specificities of each setting. However, they are linked by a common set of objectives, which focus on developing understanding of the following:

1. How have the incentive environments evolved in the shift away from conflict in each country?
2. What influenced the trajectory?
3. What have been the reform objectives and mechanisms?
4. What are their effects (intended and unintended)?
5. What lessons can be learned (on design, implementation, and suitability to context) for future interventions?

### Conceptual framework:

A conceptual framework for the research has been produced (see Figure 1). Although it is generic, it can be used to generate questions specific to post-conflict settings. For example, the context features on the left will influence attraction, retention and performance in all settings, but some will be particularly acute in post-conflict settings. In particular, the absence of actual or perceived security, the fragility of political settlements, the possibly fractured relationships with the community (or conversely, the strong ones developed during a period of loss of central control) and the breakdown of organisational controls are all hypothesised to call for different responses in the post-conflict setting. These will affect the design, implementation and impact of the general policy levers on the right (and hence the outcomes for human resources and the health system outlined at the bottom).

Figure 1 Conceptual framework, HW incentive research

All of the factors are interconnected in a dynamic relationship, and all have a potential to impact on attraction, retention and performance. The ‘policy levers’ on the right represent a range of ways in which the incentive environment can be actively engineered in some way. They include financial and non-financial measures, but this is represented as a continuum, with no hard boundaries, as some ‘non-financial’ activities such as training also have knock-on potential income effects (in the form of increased future earning potential, for example). Although the conceptual framework is presented as a cross-sectional picture, for the sake of simplicity, it is recognised that past experiences influence present expectations and behaviours. In addition, external factors will play an important part in influencing developments in relation to these various nodes. The fiscal situation and the investment strategies of donors, for example, will be an important factor enabling or constraining the different policy levers, for example.

**Research design**

In all four countries, the study design includes a substantial retrospective component. A cross-sectional survey will be carried out in two countries (excluding Cambodia and Uganda, where it would duplicate existing or ongoing work). While research will engage with national stakeholders and national datasets, some more in-depth tools will be focussed on the sites summarised in Table 1 below. In most cases, these were chosen to represent all areas of the country, with the exception of Uganda, where the Acholi sub-region is the focus of the research (as this is where 90% of displaced people are living). Some detail on the main comparison groups in the research is also included. These vary across the countries, reflecting the different contexts and research priorities.

### **Table 1 Summary of site selection, comparison groups and timeframe for research**

|  | Cambodia | Sierra Leone | Uganda | Zimbabwe |
| --- | --- | --- | --- | --- |
| Site selection | Six provinces (covering all four ecological regions) – one district from each | 2-3 sites in four districts (covering all main regions) | Three districts in Acholi sub-region – most conflict-affected area | Two provinces – one well served and one under-served |
| Significant comparison groups (in addition to cadre type) | Rural/urban; also those provinces which have received intensive external investment versus not | Primary versus secondary/tertiary level health workers (those receiving and not receiving PBF payments) | Public employment versus private not-for-profit | Staff working in government facilities, compared to those working for municipalities, rural district councils, mission sector, private |
| Timeframe | 1999 onwards (post-conflict) | 2000 onwards (last phase of conflict; post-conflict since 2002) | 2000 onwards (six years during; six years after) | 1997 onward (economic crisis, and post- since 2009) |

The timeframe for the retrospective work varies across countries. For Cambodia, the post-conflict period dates to 1993, covering almost two decades. As that is a long time-span, it will not be possible to examine data from the conflict period. We will focus on the period since 1999, when all internal conflict ended. For Sierra Leone, we will aim to capture the final period of conflict (2000-2) and the period since. For Uganda, the post-conflict period dates to 2006, but as this is recent, we will aim to compare the six years of conflict (2000-6) with the six years’ post-conflict (2006-12). In Zimbabwe, the start of the most recent period of economic crisis is dated to 1997 and we will aim to capture data from that point, and including the post-conflict period of 2009 onwards.

# Research methods

In order to answer the research questions, a mixture of qualitative and quantitative methods will be used (see Table 2). Analysis of this data, combined with lessons learned in other comparable contexts, will feed into recommendations for more effective policy responses.

### Table 2 Summary of research tools across countries

| Research tools | Cambodia | Sierra Leone | Uganda | | Zimbabwe |
| --- | --- | --- | --- | --- | --- |
| 1. Stakeholder mapping |  | √ | √ |  | |
| 1. Document review | √ | √ | √ | √ | |
| 1. Key informant interviews | √ | √ | √ | √ | |
| 1. Life histories/ in-depth interviews with HWs | √ | √ | √ | √ | |
| 1. Quantitative analysis of routine data | √ | √ |  | √ | |
| 1. Survey of health workers |  | √ |  | √ | |

## Linking tools to research questions

How policies with regard to health worker incentives have evolved during the post conflict period as well as the drivers for the policy changes will be explored through the following methods: stakeholder mapping; document review; key informant interviews; and career histories of health workers.

Challenges in implementing the health worker incentive policies and the effects of these policies (intended and unintended) will be identified and explored through the following methods, two of which focus on the health side (see Figure 2) - one more subjective, one more objective – and two of which focus on the health worker perspective – again, one being more subjective and one more quantified:

- Key informant interviews
- Analysis of trends in health worker supply, distribution and outputs over time, using routine data, disaggregated, where available, by:
  - Region, province, district
  - Different cadres such as doctors, medical assistants, nurses, midwives
  - Gender
  - Type of facility
- In-depth interviews with health workers
- Health worker survey, focussing on personal characteristics, working practices and hours, remuneration from various sources, living conditions and preferences/motivation.

### **Figure 2 How tools relate to research questions, HW incentive research**

#
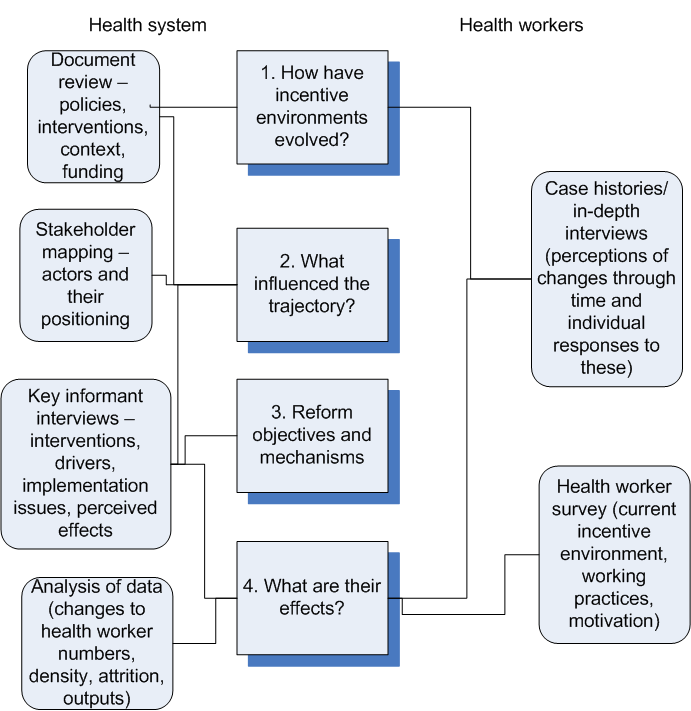


## Stakeholder mapping

*Objective*: To identify the key stakeholders who influence or are knowledgeable about human resources for health polices and their implementation.

*Approach used:* The research team will identify key informants to participate in the mapping exercise. They will be drawn from the key constituencies (e.g. donors, Ministry of Health, Ministry of Finance, professional associations, NGOs, political stakeholder) and are expected to be 4-6 in number. These individuals will be asked to brainstorm on key stakeholders in relation to human resources. Stakeholders will be placed, according to informant perceptions, along two axes (likely to be influence and interest). If possible, the immediate post-conflict and present situation may be plotted, to identify changes over time.

*Data analysis:* The mapping will generate an initial list of names of individuals, groups and organisations and this will be further developed as the study progresses. The identification of stakeholders will also help identify key informants for interviews.

*Constraints:* The stakeholders, their influence and relationships may change over time and therefore stakeholder mapping could be done at regular intervals. It will also rely on an open dynamic between participants.

## Document review

*Objective:* To describe the HRH policies, the reasons for their introduction, how they have been implemented, and any effects of the policy changes over the selected period.

*Approach used*: There is a range of documents which the research team will review which include:

- National Health Strategic Plans
- National Health Workforce Development Plan
- Mid-term review of the health workforce development plan
- Health policy interventions on HRH, such as the incentive schemes,
- Policy remuneration: salaries, allowances pensions, regulation of additional earning
- Policy documents on recruitment: placement, promotion, retirement, and training of health workers
- Documents of organisations working in HRH
- Academic studies or evaluations relating to health worker incentives.

*Data collection and analysis:* The analysis of documents will hinge around the framework of the research questions.

*Constraints:* In some contexts, there will be a considerable mass of material to abstract from; in others there may be relatively little.

## Key informant interviews

*Objective:* To understand KI perceptions of HW incentive policies, their evolution in the post conflict period, their implementation and effects.

*Sample size and sampling methodology:* KI from national down to local level will be purposively selected, according to their knowledge of the focal topics. The total number is not preset but is expected to range between 25 and 50 in total for each country.

*Data collection:* The interviews will be semi-structured and focus on the following topics:

- Challenges for HW attraction, retention, distribution and performance, before and now
- Policy iterations (whether any lessons were learnt along the way and acted on)
- Synergies or dissonances between interventions
- Implementation experiences, constraints and lessons
- Their understanding of the effects of past policies
- Current thinking on reform options and priorities.

The interviews will be tape recorded and noted after gaining permission from the participants. The interviews will take place in a private place acceptable to the interviewee, such as their office.

*Data analysis:* Thematic analysis using NVIVO will be carried out on transcribed (and sometimes translated) texts.

*Constraints:* In some countries, gaining interviews with officials can be time-consuming and access for some of the key stakeholders may be hard to gain.

## In-depth interviews/life histories with health workers

*Objective:* To explore health workers’ perceptions and experiences of their working environment, how it has evolved and factors which would encourage or discourage them from staying in post in remote areas and being productive.

*Approach used:* In-depth interviews will be conducted with health workers in selected health care facilities in the study areas using an open-ended topic guide. For life histories, older workers, near to or just past retirement, will be interviewed to understand their personal professional trajectory and what influenced it. They will be encouraged to produce visual aids, such as timelines.

*Sample size and sampling methodology:* key cadres will be defined in each country, reflecting the categories which are hardest to attract and retain in rural areas (in many cases, doctors and midwives. In selecting the doctors and midwives, gender and length of service will be considered to ensure we capture a range of responses. The overall number varies, but will be relatively low, reflecting the intensive nature of this tool – around 25 in-depth interviews as a maximum.

*Data collection and analysis:* This will be conducted in the same manner as for the key informant interviews.

The topic guide will cover the following areas:

- How they became health workers
- Their career path since, and what influenced it
- What motivates/discourages them to work in rural areas and in public service
- Challenges they face in their job and how they cope with them
- Their career aspirations
- Their knowledge and perceptions of recent and current incentives.

*Constraints:* in-depth interviewing has to be done in a sympathetic and skilled manner in order to gain maximum understanding; the same is true for the analysis stage.

## Analysis of routine data

*Objective:* To analyse trends in health worker availability, distribution, attrition, and performance during the post-conflict period.

*Approach used:* Existing human resource and selected service utilisation data will be collated from national, regional/district or facility sources (whichever are judged to be most reliable and complete).

*Data collection:* The data will be collated for the defined period using structured data extraction forms.

*Data analysis:* The data will be analysed to describe the trends in health workers supply, distribution and output during the post-conflict period. If datasets allow, some more ambitious efficiency analysis will be undertaken.

The indicators will include:

- Staffing numbers for key cadres and proportion of filled posts (where applicable)
- Staff to population ratios
- Staff to output ratios (in-patients; out-patients; combined measures; and for specific services such as midwives: deliveries, where data permits)
- Attrition rates (staff lost per year)
- Quality of care measures, where possible (e.g. fresh stillbirths).

*Constraints:* Clearly this tool relies on the completeness and accuracy of routine data sources. Where the quality is judged to be too low, analysis of relevant indicators will not be presented (or will be presented with appropriate warnings).

## Health workers’ incentives survey

*Objective:* To understand the current incentive environment facing key kinds of health workers, their characteristics and the factors which motivate and demotivate them (to provide a quantitative measure to complement in-depth interviews).

*Approach used*: A structured questionnaire will be used to collect data from defined key cadres of health workers in face-to-face interviews.

*Sample size and sampling methodology*: The study population will include all key cadres of health workers, with especial focus on those who are hard to retain. The sample size will be based on the total number of workers in each category in the selected study areas, with a smaller proportion chosen for larger groups. Sampling will be random where numbers permit. Total numbers will be in the range of 250-500 health workers.

*Data collection:* The questionnaire will focus on the following topics:

- HW characteristics
- Current earnings from different sources
- Current working patterns – public/private mix, other sources of income, dual practice etc.
- Working hours and workload
- Perceptions of working environment and factors which motivate/demotivate
- Willingness to work or stay in rural areas.

*Data analysis:* The data will be entered, cleaned and analyzed using SPSS or STATA software. Analysis will be done according to cadre, region, type of facility, and gender.

*Constraints*: The main constraint for this tool is that it relies on self-reporting. Issues of remuneration and working practices are sensitive and the fullness and reliability of responses will vary. Triangulation with other findings will be needed to establish the credibility of answers.

# Gender

Gender is important in understanding different professional roles, and in post-conflict settings there may be more acute gender constraints related to perceptions of security of postings. In this research, we will examine gender through disaggregating our routine data, where possible, and analysing how gender balance in different cadres changed over the period. We will also explore issues around gender in our in-depth interviews with health workers and our life histories.

# Ethical issues

Ethical approval for the research has been obtained from the relevant national ethical committees. The usual precautions will be undertaken to obtain informed consent, to assure confidentiality of respondents, to undertake research in a sensitive manner, and to keep data secure. This research topic deals with sensitive issues relating to pay and health worker behaviour.

There are risks in relation to personal information being overheard or passed on. These will be managed by careful choice of research location (to ensure privacy) and respecting reassurances to the respondents that all data made anonymous. This is equally applicable at higher levels, where key informants will be assured of careful presentation of findings, so that their identities are not disclosed.

# Research uptake

Strategies to maximise uptake of findings will follow the approach agreed amongst the consortium as a whole. This will include the following steps:

- Early consultation with stakeholders to establish their priorities and research needs (undertaken though the country situation analysis processes and discussions on individual research projects)
- Creating mechanisms to link with stakeholders during the course of the research so that they are engaged and more likely to be responsive to findings
- Finding appropriate channels to reach the key constituencies with research messages. For this topic, those constituencies will include not only Ministries of Health, Ministries of Finance, development partners, NGOs, but also public service commissions, local government, and professional organisations. Findings will be tailored to be clear and relevant for these different groups.

**References**

1. Hicks V, Adams. Pay and non-pay incentives, performance and motivation. Antwerp: ITG Press, 2003.
2. WHO. The World Health Report 2000—Health Systems: Improving Performance. Geneva: WHO, 2000.
3. Franco LM, Bennett S, Kanfer R. 2002. Health sector reform and public sector health worker motivation: a conceptual framework. *Soc Sci Med* 2002*;* **54:** 1255-1266.
4. Mathauer I, Imhoff I. 2006. Health worker motivation in Africa: the role of non-financial incentives and human resource management tools. *Human Resour Health* 2006*;* **4:** 24.
5. Witter S, Fretheim A, Kessy F, Lindahl A. 2011. Paying for performance to improve the delivery of health interventions in low and middle-income countries. *Cochrane Database Syst Rev* 2011; 7:CD003318
6. Lemiere C, Herbst C. Assessing the Effects of RBF Schemes on Health Worker Motivation: A gudied for RBF Impact Evaluation. AFTHE, 2011.
7. Willis-Shattuck M, Bidwell P, Thomas S, Wyness L, Blaauw D, Ditlopo P. Motivation and retention of health workers in developing countries: a systematic review. *BMC Health Serv Res* 2008; **8:** 247-247.
8. Dambisya Y. A review of non-financial incentives for health worker retention in east and southern Africa. Harare: Equinet, 2007.
9. Eichler R, Levine R. Perfomance Incentives for Global Health: Potential and Pitfalls. Washington, D.C: Center for Global Development, 2009.
10. Pavignani E. Analyzing human resources for health. Module 10. Modular manual analyzing disrupted health sectors. <http://www.who.int/workforcealliance/knowledge/resources/disruptedhealthsystem/en/index.html>. (accessed 8 october 2011)
11. O'Hanlon KP, Budosan B. Post-disaster recovery: a case study of human resource deployment in the health sector in post-conflict Kosovo. *Prehosp Disaster Med* 2011; **26:** 7-14.
12. Shuey DA, Qosaj FA, Schouten EJ, Zwi AB. Planning for health sector reform in post-conflict situations: Kosovo 1999-2000. *Health Policy* 2003; **63**: 299-310.
13. WHO. Guide to health workforce development in post-conflict environments*.* Geneva: World Health organsiation, 2005.
14. Nelson BD, Simic S, Beste L, Vukovic D, Bjegovic V, Vanrooyen MJ. Multimodal assessment of the primary healthcare system of Serbia: a model for evaluating post-conflict health systems. *Prehosp Disaster Med* 2003; **18:** 6-13.
15. Macrae J, Zwi AB, Gilson L. A triple burden for health sector reform: 'post'- conflict rehabilitation in Uganda. *Socl Sci Med* 1996; **42:** 1095-1108.
16. Varpilah ST, Safer M, Frenkel E, Baba D, Massaqui M, Barrow G. Rebuilding human resources for health: a case study from Liberia. *Human Resour Health* 2011; **9:** 11-11.
17. Sirkin S, Cali SF, Keough ME, Levy BS, Sidel VW. The Roles of Health Professionals in Postconflict Situations. *War and Public Health.* Oxford: Oxford University Press, 1999.
18. Pavignani E. Human Resources for Health through Conflict and Recovery: Lessons from African countries. *Disasters* 2011.
19. Martins N, Kelly PM, Grace JA, Zwi AB. Reconstructing Tuberculosis Services after Major Conflict: Experiences and Lessons Learned in East Timor. *PLoS Med* 2006; **3:** 1765-1775.
20. Hill P. Ethics and Health Systems Research in 'Post'-Conflict Situations. *DevWorld Bioeth* 2004; **4:** 139-153.
21. Macrae J. Dilemmas of ‘post’-conflict transition: lessons from the health sector. *Relief & Rehabilitation Network, Network Paper No. 12*., 1995.
22. Smith J. Human resource development in East Timor. Assignment Report WHO: IR/TMP/EHA/021. Geneva: World Health Organsiation, 2001
23. High-Level forum on the Health MDGs. Health Service Delivery in Post-Conflict States. 2005.
24. Newbrander W, Yoder R, Debevoise AB. Rebuilding health systems in post-conflict countries: estimating the costs of basic services. *Int J Health Plann Manage* 2007; **22:** 319-336.
25. United Nations. *Reconstructing Public Administration after Conflict. Challenges, Practices and Lessons Learned*. New York: United Nations, 2010.
26. Glenton C, Scheel IB, Pradhan S, Lewin S, Hodgins S, Shreshtha V. The female community health volunteer programme in Nepal: decision makers' perceptions of volunteerism, payment and other incentives. *SocSciMed* 2010; **70:** 1920-1927.
27. Buchan J. What difference does ("good") HRM make? *Human Resour Health,* 2004; **2:** 6.
28. Lehmann U, Dieleman M, Martineau T. Staffing remote rural areas in middle- and low-income countries: A literature review of attraction and retention. *BMC Health Serv Res,* 2008; **8:** 19.
29. WHO. Increasing access to health workers in remote and rural areas through improved retention. Global Policy Recommendations. Geneva: World health Organsiations, 2010.
30. WHO. World Health Report. Health Systems Financing. The path to universal coverage. Geneva: World health Organsiations, 2010.
31. Toonen J, Canavan A, Vergeer P. 2009. Performance Based Financing: a synthesis report. Amsterdam: KIT, 2009.
