# Supplementary material for: What adaptation to research is needed following crises: a comparative, qualitative study of the health workforce in Sierra Leone and Nepal
Source: Health Res Policy Syst. 2018 Feb 7;16:6. doi: 10.1186/s12961-018-0285-1 (PMC5804047; doi:10.1186/s12961-018-0285-1)
Supplement: Supplementary file 2 — Sierra Leone adapted study protocol. (DOCX 59 kb) [file 12961_2018_285_MOESM2_ESM.docx]

# Additional File 2: Sierra Leone adapted study protocol

**Ebola in Sierra Leone: effects on health workers and the health system and lessons for future health system reconstruction**

## Background

The 2014 Ebola Virus Disease (EVD, or “Ebola”) outbreak continues to evolve in alarming ways in Sierra Leone. Currently the virus has spread to all 14 districts and the country is still struggling to control the escalating outbreak against a backdrop of severely weak health systems and significant deficits, with reports of five new cases per hour in Sierra Leone and an alarming prediction of 1.4 million people infected in both Sierra Leone and Liberia, if efforts to control the spread of the virus are fruitless. The health system in Sierra Leone was already weak and over-burdened prior to the Ebola outbreak. The health system has now been further weakened by the outbreak and all efforts made in the post conflict era to strengthen the health system and working towards providing equitable access to health care for all has suffered a major setback. Thus, in the recovery phase, it is imperative that efforts should be made to rebuild the current crumbling health system. In Sierra Leone the health system is divided into the six pillars,^1^ and the outbreak has highlighted the gaps and challenges in these pillars which contributed to the delayed response. Some of these challenges are highlighted in the figure 1.

**Figure 1: Pillars of the Health sector in Sierra Leone and challenges associated with each pillar**

There is need for health systems research in Sierra Leone to better understand how to strengthen the six pillars and build the foundation for a responsive and resilient health sector working towards providing universal health coverage for all.^2,3^ A recent paper highlighted that a weak health system cannot be resilient and cope with crises such as an Ebola outbreak, and called for “national governments, assisted by external partners to develop and implement strategies to make their health systems stronger and more resilient” (page 850).^4^ ReBUILD’s research into health system development post-conflict in Sierra Leone can provide a good historical basis for understanding the effects of this latest shock on the health system and for developing lessons for post-Ebola reconstruction.

# *Human resources for health*

The health systems pillars are interconnected and serve as the foundation to having a responsive and resilient health sector in the face of a health crisis such as the current Ebola outbreak. One of the most important pillars of a responsive and effective health system is having adequate numbers of skilled human resources.^5^

Understanding the post-conflict dynamics of the HRH in Sierra Leone was the basis of the ReBUILD research consortium’s ‘health worker incentives in Sierra Leone’ project. Findings from this study has highlighted that building the capacity of health workers and developing a motivated health work force is an ongoing issue.^6^ In the post conflict era, efforts had been made to strengthen the HRH pillar within the health care delivery sector with a number of HRH reforms implemented.^7^ Analysis of routine available data showed an increase in the numbers of health workers, due to a rapid recruitment drive and salary increase in the run up to the introduction of the Free Health Care Initiative. This had a positive impact on retention, even in the hard to reach areas of Sierra Leone. The data also showed improvements in absenteeism after the implementation of the Staff Sanction Framework and its accompanying attendance monitoring tool.^8^

However, efforts made in the post conflict period to strengthen HRH has suffered a major knock by the current outbreak of Ebola.^9^ Health workers in general are demotivated even before the outbreak. Health facilities are chronically understaffed by poorly trained, overworked healthcare personnel, with very little or no training on infection control practices to deal with the outbreak. Working conditions are generally poor, lacking adequate logistics. The basics of sanitation, electricity and personal protective equipment to ensure the safety of health workers from infection are not always available when required. This has resulted in the majority of health specialists at the forefront of the outbreak being international experts flown in by international development partners, highlighting the heavy dependence on foreign expertise. As of the 5^th^ of November 2014, 102 national health workers have died including specialist doctors which further depletes the already inadequate health workforce.^10^ With only 2 medical doctors per 100,000 of the population in Sierra Leone (before the Ebola outbreak), the human resource available for health is inadequate, which poses a critical constraint to sustainable development. Without an adequately trained and appropriately distributed health workforce, Sierra Leone cannot run an effective and responsive health system or meet the basic requirements of access to essential health care services for all. Annual attrition rates for health workers ranged from 9 to 50% for different cadre of medical staff in 2011, i.e, even before the outbreak.^8^ This highlights the challenges in the HRH sector in the pre-Ebola phase that have been further exacerbated during the current outbreak. Understanding these challenges should be an integral part of the response in the post Ebola phase. The report by Ulrich^11^ gives insight into the additional burden of ‘moral distress’ experienced by health workers, during the ongoing Ebola crisis. The writer describes this as ‘loss of professional integrity’ that can have emotional consequences. Local health workers are inadequately equipped to support all the immediate needs of Ebola infected patients, become overwhelmed with the emotional nature of the work. With a number of their colleagues succumbing to the virus, some are faced with making a moral judgement between continued service and deal with death on a constant basis or ‘conscientious objection’ to work in order to protect themselves and their families.

Financial and non-financial incentives for health workers is just as important as ensuring that enough human resource for health is available to provide equitable health care service. However, the commitment and performance of the health workforce, depends on several factors including but not limited to the following: leadership and governance, conditions of service, continuing professional development, enabling working environment for effective service delivery, and provision of incentives.^6^ In addition, for the scope of this study, challenges pertaining to leadership and governance and service delivery and their impact on health worker performance will be explored.

## *Service delivery*

Poor infection control and lack of disease control and prevention units in the health facilities compromise the basic package for essential health service in Sierra Leone. There is also an element of mistrust between service users and health workers, which can only intensify if the spread of the virus continues to pick up momentum. Health workers were ill equipped to deal with Ebola patients and with their colleagues succumbing to the disease they are afraid to go to work. This has resulted in decreased service utilisation and severe disruption to provision of services such as maternal and child services as well as health care programmes. In addition, the enablers for effective and efficient functionality of the health care delivery sector in terms of infrastructure and infection control are absent. These include but not limited to: pipe borne water, sanitation, sustained electricity supply countrywide, safe means of disposing of clinical waste and suitable infrastructure to house isolation and treatment centres. The impact of the absence of these enablers on health worker performance should also be elucidated further.

## *Leadership and governance*

Decentralisation of the health system in Sierra Leone followed the Local Government Act of 2004 which established 19 Local Councils that are responsible for managing primary health care services. These local councils are made up of 13 district and 6 city councils. Each of the 13 districts has one district council. In addition, Bo, Bonthe, Kenema, Koidu and Makeni towns, and Freetown city each has one city council. There are 13 District Health Management Teams (DHMTs), seven covering one district council each and six covering one city and one district council each.The DHMTs in collaboration with other key stakeholders are in charge of primary health care service delivery. They plan, organise, manage, implement, monitor and supervise health programmes in their districts. The DHMTs make yearly plans which are presented to the Ministry of Health and Sanitation (MoHS) for funding. Tertiary and secondary health care are being devolved to the local councils. However, the outbreak has highlighted that the decentralisation process is not implemented effectively, as Central Government still makes the decisions in the health sector. Delayed or poor decisions from Central Government as seen in the current outbreak, affected district level responsiveness. There were critical delays in the response to the current outbreak in terms of procurement of supplies and drugs to equip health workers adequately in the health facilities.

## Demand for the research in Sierra Leone

There was a delayed response to the current outbreak due to a number of factors ranging from governance and leadership, lack of readiness in the health facilities and challenges pertaining to the human resources for health. Therefore there is a need to understand how the health system responded to the current Ebola crisis, from a health workers perspective. There is a need to unpick the factors that supported or hindered health workers’ ability to cope with the crisis. There is an urgent need to generate findings that can be fed into the ongoing crisis as an immediate response, and also generate findings that can be utilised in rebuilding the crumbling health sector in the post- Ebola phase, and for any further outbreaks of this nature as a longer term response.

## Aim and objectives of the research

### *Aim*

To understand the challenges to a responsive and resilient health system from a health worker perspective in the face of the recent Ebola shock, and how to build resilience to such shocks in the future

### *Objectives*

1. To understand health workers’ experiences of the Ebola crisis, and how it has added to previous challenges which they faced
2. To explore the factors which helped some to cope, while others were unable – personal as well as systemic factors
3. To develop recommendations based on reinforcing these coping strategies and supportive systems

# Study design

This is a retrospective cross sectional study using qualitative research methods. The timeframe for the retrospective data collection will be from the pre Ebola outbreak in 2013 to the present day.

In order to meet the stated objectives, the study will employ different methods and data collection tools:

- Document review to include tracking of reports by Development Partners, Ebola Response Team and Ministry of Health and Sanitation on trends in health service coverage and staffing levels pre and during Ebola, as well as relevant online publications on the Ebola crisis and its effects on health workers and the health system in Sierra Leone
- Key informant interviews with district level managers including DHMT members, Local Council members, health facility managers and development partners
- In depth interviews with frontline health workers including national and international health workers working in Ebola treatment centres and national health workers working in other health facilities

## Study sites

The Ebola outbreak has affected all the districts in Sierra Leone, some more than others. Therefore, the rationale for the selection of districts has focused on ensuring a range of different types of districts using the criteria of rural and urban populations, hard to reach areas and poverty. Four districts are selected from the four regions of Sierra Leone. In each district, the district hospital and two health centres will be selected. The selected study districts are:

1. Western Area (Urban/Rural) District – new epicenter
2. Kenema District (Eastern Region) – former epicenter
3. Bonthe District (Southern Region) – low
4. Koinadugu District (Northern Region) – recently hit, no treatment centre, hard to reach

# Methods

## Method 1: Document Review

*Objective*

- To synthesise existing materials on how the Ebola crisis has affected services in the districts, the health system and health workers in particular
- To analyse the trends in health service coverage from 2013 (pre Ebola) to the present day.
- To analyse the trends in health worker distribution and attrition from 2013 (pre Ebola) to the present day

*Data collection and analysis:*

The research team will access secondary data from the MoHS, documents from government departments and their websites, and other organisations involved in evaluating the performance of the health sector such as health sector performance report and other online reports. The documents will be reviewed by the research team to answer the key questions:

1. How have the pre-existing challenges faced by the health workforce contributed to the spread of the virus and been affected by it?
2. What were the effects of Ebola on service delivery and utilisation and the health system as a whole in these districts?
3. What is the correlation between health service coverage and output and health worker distribution pre and post Ebola?
4. What is the attrition of health workers pre and post Ebola? What are the reasons for attrition?

*Constraints*

Data on service coverage and health facility staffing may not be available. Donor documents may be difficult to obtain and may even be confidential.

## Method 2: In depth interviews with frontline health workers

*Objectives*

- To explore health workers’ perceptions and experiences of the ongoing Ebola crisis in Sierra Leone and the impact of the Ebola outbreak on health workers
- To identify any facilitators, constraints, challenges and coping mechanisms, in relation to leadership and governance, HRH and service delivery
- To explore options to increase the resilience of workers and the health system as a whole in future

*Sampling methodology and size*

In-depth interviews will be conducted with a range of workers (including staff within the public health system and international development partners namely Save the Children, IRC and King’s Partnership, as they are in operation in our study sites), who have been involved with the treatment/management of Ebola patients. These will include health workers who already participated in the ReBUILD HRH project to understand how they have been affected by this next shock in the health system.

There will be four groups of participants selected for these interviews. Table 1 provides details of the sampling by district:

1. Health workers who were interviewed for the ReBUILD HRH study: we will follow up as many as possible of the 23 participants included in the HRH study. It is anticipated that some may have died, others may have left the country, some will be working in the Ebola treatment or isolation centres, some will be working in other health facilities, and others may not be working in frontline health services. They will form a mini-cohort group for follow up. Even when not available for interview, we will try to document their current status, where possible.
2. National health workers working in Ebola treatment or isolation centres: we will select 2 health workers working in each centre in the most hit study districts, Western Area and Kenema. These may be included in the participants described in group 1, but if not then we will purposefully select from the centres.
3. National health workers working in other health facilities: we will select 2 health workers working in a district hospital and community health centre in each study district. These may be included in the participants described in group 1, but if not then we will purposefully select from the health facilities. This group will allow us to understand the wider effects of Ebola, beyond the specific treatment centres.
4. International health workers working in Ebola treatment or isolation centres: we will select international health workers working in the Ebola treatment or isolation centres in the most hit study districts, Western Area and Kenema. A maximum of 4 health workers will be interviewed in order to capture the perceptions of outsiders with operational insights on the current functioning of service delivery in the districts.

Health workers (both local and international) will be selected from government led and international partner led Ebola centres in the Western Area and Kenema. In the Western Area health workers will be selected from the Government led Police Training School (PTS) treatment centre in Hastings, Kerry Town treatment centre (run by Save the Children) and from the isolation centre at Connaught Hospital supported by King’s Partnership. In Kenema, they will be selected from the Government led Kenema Government Hospital.

**Table 1: Sampling for in depth interviews with health workers**

| **District** | **HRH study cohort** | **National health workers in health facilities** | **National health workers in Ebola centres** | **International health workers in Ebola centres** | **Total** |
| --- | --- | --- | --- | --- | --- |
| Western Area | 11 | 0-2 | 0-2 (one each from Hastings and Connaught) | 2 (one each from Kerry Town and Connaught) | 17 |
| Koinadugu | 5 | 0-2 | N/A | N/A | 7 |
| Kenema | 4 | 0-2 | 0-2 (Kenema Government Hospital) | 2 (Kenema Government Hospital) | 10 |
| Bonthe | 3 | 0-2 | N/A | N/A | 5 |
| **Total*** | **23** | **8** | **4** | **4** | **39** |

*totals will be lower as there will be overlap between national health workers and HRH cohort

*Data collection*

The research team will conduct the in depth interviews face to face or by telephone, depending on the situation at the time of the interview with regard to travel, safety and workload of health workers, as well as agreement with the interviewee. Where possible we will conduct the interviews face to face as this will enable for more in-depth exploration and probing of the key issues, as well as allow the interviewer to gauge the reactions of the participants to being interviewed. The team leader will facilitate the interview using a topic guide whilst the research assistant takes notes and observes the process. The interviews will be tape recorded after gaining permission from the participants. The interviews will take place in a private room in the health facility or in their home whichever is acceptable to the participant. It is anticipated that most of these interviews will be conducted in English. However, the interview will be conducted in the local language if requested by the informant.

*Data analysis*

The recordings will be transcribed verbatim. Where necessary, the recordings will be transcribed in the local language then translated into English. Back translation from English into the local language will be conducted in a small sample of transcripts to assure accuracy of the translation. The interviews will be analysed inductively through thematic analysis.^12^ Participants’ responses will be coded and grouped by themes related to the research questions. Qualitative analysis software, NVivo will be used to help manage and analyse the data.

*Constraints*

Although many efforts will be made to ensure the quality of the translation and transcription, it is inevitable that some data will be lost. During the telephone interviews it will be difficult to explore the issues in depth, and assess the reactions of the participants.

## Method 3: Key Informant Interviews

*Objectives*

- To explore key informants’ perceptions and experiences of the Ebola outbreak, in particular the impact on health workers
- To identify the constraints, challenges and opportunities in relation to leadership and governance, HRH and service delivery during the Ebola outbreak
- To explore options to increase the resilience of workers and the health system as a whole in the post Ebola phase

*Sampling methodology and size*

The key informants will be purposefully selected for inclusion based on them being a member of the DHMTs, local councils, health facility managers and international partners working in the study districts. They will therefore have a detailed knowledge of the health system response to the current outbreak and be able to provide their perceptions and experiences of the response. The number of key informant interviews is estimated to be in the region of 20 (Table 2).

**Table 2: Sampling for key informant interviews**

| **District** | **DHMT** | **Local Council** | **Health facility manager** | **International partner** | **Total** |
| --- | --- | --- | --- | --- | --- |
| Western Area | 1 | 1 | 2 (district hospital; community health centre) | 1 (King’s Partnership) | 5 |
| Koinadugu | 1 | 1 | 2 (district hospital; community health centre) | 1 (CARE International) | 5 |
| Kenema | 1 | 1 | 2 (district hospital; community health centre) | 1 (International Rescue Committee) | 5 |
| Bonthe | 1 | 1 | 2 (district hospital; community health centre) | 1 (World Vision) | 5 |
| **Total** |  |  |  |  | **20** |

*Data collection*

The research team will conduct the key informant interviews face to face or by telephone, depending on the situation at the time of the interview with regard to travel, safety and workload of informants, as well as agreement with the interviewee. Where possible we will conduct the interviews face to face as this will enable for more in-depth exploration and probing of the key issues, as well as allow the interviewer to gauge the reactions of the participants to being interviewed. The team leader will facilitate the interview using a topic guide whilst the research assistant takes notes and observes the process. The interviews will be tape recorded after gaining permission from the participants. The interviews will take place in a private room, for example, in their office. It is anticipated that most of these interviews will be conducted in English. However, the interview will be conducted in the local language if requested by the informant.

*Data analysis*

The recordings will be transcribed verbatim. Where necessary, the recordings will be transcribed in the local language then translated into English. Back translation from English into the local language will be conducted in a small sample of transcripts to assure accuracy of the translation. The interviews will be analysed inductively through thematic analysis.^12^ Participants’ responses will be coded and grouped by themes related to the research questions. Qualitative analysis software, NVIVO, will be used to help manage and analyse the data.

*Constraints*

Although many efforts will be made to ensure the quality of the translation and transcription, it is inevitable that some data will be lost. During the telephone interviews it will be difficult to explore the issues in depth, and assess the reactions of the participants.

# Gender

In the analysis, we will explore the gendered themes in relation to challenges faced by health workers during the Ebola outbreak and their coping strategies.

# Ethics

Applications for ethical approval will be made to the Sierra Leone Scientific and Ethics Committee and the Liverpool School of Tropical Medicine Research Ethics Committee prior to the commencement of the study.

Interviews with health workers and managers:

- There is potential for causing distress during these interviews. The interviews will be conducted by a Sierra Leonean researcher who has been working with health workers during the Ebola outbreak. She is therefore very sensitive to the complex situation and will be able to ask questions and respond to the health workers in a considerate and caring manner. She will conduct the interviews face to face or by telephone, depending on the situation at the time of the interview with regard to travel, safety and workload of informants, as well as agreement with the interviewee. Where possible we will conduct the interviews face to face as this will enable for more in-depth exploration and probing of the key issues, as well as allow the interviewer to carefully assess how the interviewee is coping with the interview and can stop the interview at any time. We will also organise, where possible, counselling sessions for participants who may require more support.
- Health workers’ and managers’ time to participate in the study: we recognise that we will be taking up valuable time that could be used treating patients. We will therefore find a time that is convenient for the health workers and managers and does not disrupt service provision.

Research team:

- Protection from infection: the SL project lead researcher is a trainer at the DfID Ebola training academy in Sierra Leone, training health workers in effective infection control measures and how to work safely in an Ebola treatment unit. Other precautionary measures will include checking the temperature of the study participant, washing hands regularly and avoiding bodily contact.
- There is also potential for distress for the researchers when they hear and witness the challenges faced by health workers. Support for the researchers will be provided through: a field work plan that allows for enough time between interviews, regular debriefing sessions amongst the Sierra Leone team, regular skype calls with the UK research team, and organising counselling if necessary.

# Research uptake

At the start of the study, meetings will be held with key stakeholders in the Ministry of Health and the frontline international partners to build awareness of the study including the concept, purpose and approaches to be used. Towards the end of the study, the preliminary results will be discussed with selected stakeholders, before they are shared with the wider community of stakeholders. Policy briefs and other documents will be developed. To ensure that the results of this study will be used by the policy makers and key influential stakeholders, a broad plan for research uptake will be implemented.

**References**

1. Government of Sierra Leone. National Health Sector Strategic Plan 2010 – 2015. Freetown: Ministry of Health and Sanitation, 2009.
2. Wurie H. Ebola’s collision with the Sierra Leone post-conflict health system <http://www.healthsystemsglobal.org/GetInvolved/Blog/TabId/155/PostId/27/ebolas-collision-with-the-sierra-leone-post-conflict-health-system.aspx> (accessed 5 January 2015)
3. Wurie H. An update from the frontline of health systems research in Sierra Leone <http://rebuildconsortiumconnect.wordpress.com/2014/10/30/an-update-from-the-frontline-of-health-systems-research-in-sierra-leone-by-dr-haja-wurie/> (accessed 5 January 2015)
4. Kieny MP, Evans DB, Schmets G, Kadandale S, 2014. Health system resilience; reflections on the Ebola crisis in West Africa. *Bull World Health Organ* 2014; 92: 850.
5. WHO. World Health Report. Geneva: World Health Organisation, 2006.
6. Wurie H, Witter S. Serving through and after conflict: life histories of health workers in Sierra Leone. <http://www.rebuildconsortium.com/publications/documents/IDIreportSLfinal230614.pdf> (accessed 8 January 2015)
7. Bertone M, Samai M, Edem-Hotah J, Witter S. A window of opportunity for reform in post-conflict settings? The case of Human Resources for Health policies in Sierra Leone, 2002-2012 *Conflict Health* 2014; 8:11.
8. Wurie H, Samai MH, Witter S. Staffing the public health sector in Sierra Leone, 2005-11: findings from routine data analysis. <http://www.rebuildconsortium.com/publications/index.htm> (accessed 8 January 2015)
9. Witter S and Wurie H. Health workers are in the front line but should not be at the back of the queue after Ebola. <http://blogs.lshtm.ac.uk/hppdebated/> (accessed 10 January 2015)
10. Ulrich C M. Ebola Is causing moral distress among African healthcare workers. Br Med J 2014; 349: g6672.
11. WHO. Ebola Response Roadmap Situation Report 5^th^ November 2014

<http://apps.who.int/iris/bitstream/10665/137510/1/roadmapsitrep_5Nov14_eng.pdf?ua> (accessed 10 January 2015)

1. Ritchie J, Spencer L, O’Connor W. Carrying out qualitative analysis. In: Ritchie J, Lewis J, eds. *Qualitative Research Practice: A guide for social science students and researchers.* London: Sage Publications, 2003: 219-262.
