# Supplementary material for: What adaptation to research is needed following crises: a comparative, qualitative study of the health workforce in Sierra Leone and Nepal
Source: Health Res Policy Syst. 2018 Feb 7;16:6. doi: 10.1186/s12961-018-0285-1 (PMC5804047; doi:10.1186/s12961-018-0285-1)
Supplement: Supplementary file 3 — Nepal original study protocol. (DOCX 209 kb) [file 12961_2018_285_MOESM3_ESM.docx]

**Additional File 3: Nepal original study protocol: Empowering district-level managers to improve Health Worker Performance in Nepal**

# Project summary

A remarkably effective initiative to improve health worker performance was developed by a District Health Officer in a hill district (Doti) of Nepal in 2009. This study will generate further understanding of this model and replicate an improved version of the model it in different ‘intervention’ contexts (mountain, hill, and plains districts). A multi-method process evaluation will be used to document the development and implementation processes involved, assess effectiveness and scale-up feasibility.

*Aim:* To assess the processes and effectiveness of a health workforce performance model (HWPM) in improving staff performance and health outcomes and its scalability in Nepal

*Objectives:*

1. To identify lessons from Doti District pilot intervention on improving effectiveness and efficiency of health workers and develop an understanding of what worked, how and why;
2. To analyse the context in the 3 intervention districts (mountain, hill and plains) and ascertain implementation issues and strategies
3. To develop, refine and test a HWPM model in 3 different districts, assess processes, effectiveness and scalability

*Intervention:* The key components of the HWPM in Doti included: group monitoring and assessment; individual appraisal; supportive supervision and feedback; development of outcome focused job aid of health workers, and community assessment. We will conduct a process evaluation of the HWPM experience in Doti, followed by a contextual analysis of intervention districts (3). An interactive workshop with policy makers, district staff, community and health professional organisations, informed by the process evaluation of the pilot, contextual analysis in the 3 intervention districts and a review of international experience of similar initiatives, will be used to develop a contextually feasible HWPM. Following orientation of district managers the improved HWPM will be implemented in the 3 intervention districts.

*Methods:* This is an implementation research, using a mixed method process evaluation. Qualitative approaches will include in-depth interviews, focus group discussion, ethnographic observations, and participatory observation techniques. These techniques, together with an analysis of relevant routine health service data, will generate an in-depth understanding of the context, implementation processes and changes in context during implementation in districts and at national level. Analysis of data through time, including base-line and endline data collection will support further understanding of implementation issues. Changes in staff motivation will be measured before and after the intervention using a tool which will be adapted to the Nepal context following the Doti process evaluation for assessing health worker motivation and satisfaction drawing on existing validated tools.^1,2^ Baseline quantitative data on health service delivery outcome indicators will be collected using record review of district-level facility data; implementation evaluation (inputs and outputs) and their relevance with respect to attaining desired outputs; in-depth understanding of changes in outputs (what worked or otherwise and why and how); and detailed documentation of overall factors that facilitated and hindered the intervention, and plausibility of scalability of the intervention will be documented.

*Study site:* Purposive selection of 3 districts, one from each ecological zone (mountain, hill and plains).

*Expected Outcomes:*

*Understanding what works where, why and how:* A tested and improved HWPM, documented understanding of the processes involved in the HWPM in Doti and the 3 different implementation districts; a HWPM implementation guide and training manuals

*Anticipated effectiveness outcomes:* increased motivation of health care workforce delivering quality health care services, increased functionality of health facility and reduction in staff absenteeism*;* Improved health service delivery outcomes (i.e. maternal and child health)

*Tentative time frame:* 24 months in total: process evaluation of the pilot and contextual analysis of implementing district 1-6 month; develop materials and tools for intervention package 7-8 month; intervention and process evaluation 9-20 month; final evaluation and write-up 21-24 month.

# Detailed description of the project

**Background information and rationale**

A well-performing workforce is described by WHO as one in which staff are available, competent, responsive and productive.^3^ Several studies have identified limitations within Nepal’s health workforce in all of these aspects. For example, health facility surveys have shown that only 64-80% of doctors, 68-81% of nurses and 81-92% of paramedics are available for work.^4^ These Human Resource (HR) problems have a direct impact on health service delivery, particularly maternal and new born services, e.g. the lack of staff to support safe delivery in government facilities was not due to the lack of trained staff but the inability to attract and retain them.^5^ The impact of supervision has also been assessed in a sample of districts and only found to have a minimal impact on health service delivery,^6^ hence lack of supportive supervision continues to be an issue. Recent capacity assessments of the health sector workforce have identified the need to strengthen leadership and management.^7^ Studies have found that HR issues – recruitment, deployment, retention and motivation – are very much context specific and vary within mountain, hill and Terai (plains) districts. A further limitation in the development of leaders is highlighted by the observation of the “drifting away of senior female staff across the health sector”.^8^ The lack of opportunities for career progression affects women across Nepal’s health sector and serves to de-motivate female staff.

Practical experience aligns with these studies, the Director of National Health Training Centre (collaborator on this study) who developed the pilot in Doti, and other DPHOs have identified the following issues from workforce assessments and from the direct experience:

- High turn-over and poor retention of staff.
- Lack of job descriptions, and when they do exist, they tend to be based on tasks rather than overall outcomes.
- Limited supervision, particularly supportive supervision that is effective in improving health workforce performance hence health outcomes.
- Lack of context specific action planning to resolve HR performance problems when identified
- Lack of accountability to communities.

Much of the evidence of what works in improving workforce performance is based on the understanding of the complexities of the factors influencing staff.^9,10^ These complexities require integrated HR strategies, described by Buchan as “linked and coordinated HRM interventions [that are] more likely to achieve sustained improvements in organisational performance than single or uncoordinated interventions”.^11^ WHO recommends considering HRM in conjunction with other key elements of the health system, particularly health information, health financing, leadership and governance, and access to essential medical products, vaccines and technologies and health services.^9^

Output 3 of the recent Human Resource for Health (HRH) Strategic Plan focuses on the improved performance of health workers and emphasises the need to strengthen performance related supervision, particularly through the development of job descriptions and review processes. The HRH Strategy provides a valuable and timely opportunity for testing the proposed intervention as MoHP is looking for evidence of the effectiveness of interventions that can be scaled up.

The intervention in this study was developed by a District Public Health Officer (DPHO) (now Director of NTHC as above), based on international HRM evidence, but applied and simplified to fit the health system context in Nepal. The intervention consisted of: group monitoring of health facility and feedback; supportive team and individual supervision, outcome focused revised job aid, peer evaluation and mentoring. Initially developed in Doti District (Far West), the HR strategies tried appeared to have had a positive impact on maternal mortality, with increases in births attended by an Skilled Birth Attendant (SBA) (from 11.44% in 2009/10 to 22.7% in 2010/11 DoHS) and in the proportion of women having 4 Antenatal Care (ANC) visits (29.77% in 09/10 and 43.95% in 10/11). The extent to which this intervention is appropriate to other districts in Nepal and the processes and costs involved are not yet known. With the publication of the HRH Strategy 2012, testing an intervention that shows promise in improving health workforce performance and thus health outcomes, is both timely and necessary. We will adapt the intervention to fit the realities of 3 different districts in Nepal and document processes, costs and its scalability.

##

## Study goal and objectives

Goal: A health care workforce performing effectively and efficiently to deliver quality health services

Aim: To assess the processes and effectiveness of a health workforce performance model (HWPM) in improving staff performance and health outcomes and its scalability in Nepal

*Objectives:*

1. To identify lessons from Doti District pilot intervention on improving effectiveness and efficiency of health workers and develop an understanding of what worked, how and why;
2. To analyse the context in the 3 intervention districts (mountain, hill and plains) and ascertain implementation issues and strategies; and
3. To develop, refine and test a HWPM model in 3 different districts, assess processes, effectiveness and scalability

*Innovation being tested:*

The innovation was developed by District Public Health Office (DPHO) in Doti district where it proved to be successful in increasing health outcomes. Adapting and extending the approach to 3 more districts will allow it to be tested in different contexts and gain an in-depth understanding of feasibility to scalability.

The key components of the intervention are as follows:

Table 3: Key components of the intervention

| **Strategy** | **What does it involve** | **Purpose** |
| --- | --- | --- |
| **1. Institutional Level: Group Monitoring and action planning** | Initially bi-annually and subsequently annually, a team of peers visit health facilities and grade them against routine indicators, particularly those on maternal and child health and aspects of health worker performance such as absenteeism, regularity of appraisals and training undertaken. The facility appraisal is conducted in the morning and in the afternoon an action-planning workshop is held for facility staff to identify practical solutions that they can do to improve any of the issues identified in the monitoring visit. (DPHO, HFMC, FCHVs and other HF staff) | To recognise good practice and motivate those on the monitoring group to learn and try similar approaches in their own facilities.  To identify poor practice and pragmatic ways of addressing these.  To establish a sense of supportive competition between health facilities. |
| **2. Community Assessment** | Community assessment of facilities and action planning are an integral part of the group monitoring exercise. During the peer monitoring visit, officers from DPHO and members of the HFMCs conduct rapid participatory appraisal PRA exercises with communities to identify the positive aspects of the health facilities and the areas they wish to improve. This community feedback is then presented along with the group monitoring feedback to the facilities’ action planning workshop.  During the workshop the facility staff and the HFMCs are facilitated by the group monitoring team to identify specific actions they can undertake to improve their health facility. The emphasis is on the actions being achievable by the clinic staff themselves, rather than requiring external financial or other assistance. | To improve accountability and ensure staff feel motivated to provide a service that meets the needs of their communities.  If positive feedback is received this can motivate staff.  If negative feedback is received this can encourage staff to make changes so as not to appear to be letting their communities down. |
| **3. Supportive supervision and feedback** | Health workers are appraised regularly (at least annually) by their colleagues. Each HW is appraised by four other staff: 1 from the DPHO, 1 colleague of the same professional level from outside the health facility and from the same level HF and 2 colleagues from within the health facility (normally one will be the in-charge). Individual workplans in line with the targets for the health facility will be set. While this intervention goes against the hierarchical relationships found within Nepal’s health workforce, it has been seen to work within Doti district particularly with a strong lead from the DPHO, who clearly endorsed the approach. | To reduce bias which may occur when appraisals are done by only one manager, normally the in-charge.  To encourage professional development through peer support  To link performance review with action plans to improve performance in line with facility targets. |
| **4. Outcome focused Job Descriptions:** | Managers at District and Facility level are trained to review job descriptions and to rewrite these in a participatory way with their staff to address targets for their health facilities.  Staff are facilitated by managers to write their own job descriptions. These job descriptions are to focus on the targets and outcomes to be achieved rather than the tasks to be completed. | To ensure all health workers are clear on their roles and responsibilities  To build a sense of purpose among health workers  To build the leadership and management skills of in-charges (facility line managers) and district managers. |

The diagram below illustrates how the inputs, outputs and outcomes aim to not only improve workforce motivation and performance, but also how the close engagement with policy makers in National, Regional and District level will inform the development of HR systems across Nepal. This proposal comes at a time of great opportunity for policy change and influencing practice, due to the recent approval of an HR strategic plan and the development of the 3rd Nepal Health Sector Programme (NHSP). This process is illustrated below:

**Figure 1: Impact of the Study at District and National Level**

**Context:** low motivation of health workforce, non-availability of staff, absenteeism, lack of supportive supervision and feedback, poor performance appraisal system, high turnover and retention of health workers, unclear job aid and unfocused, lack of accountability to community

**OUTPUTS**

- Process evaluation report from the pilot district with documented lessons of what worked, how and why
- Context analysis report of 3 intervention districts (baseline information - qualitative and quantitative) with a detailed analysis of the context in mountain, hill and plain districts and understand implementation issues and strategies
- Final intervention model and implementation guide
- Trained and skilled district managers and supervisors, health workers and health facility management committee members to implement the model
- Proper documentation of operational feasibility and constraints of the model (what worked or not, why and how)
- Greater understanding of scalability of the model - feasibility, cost (additional)
- Ownership and acceptability fostered in implementing districts

**OUTCOMES**

**NATIONAL LEVEL:**

- A tested and improved costed HWPM supported by an implementation guide and training manuals with documented understanding of processes involved

**DISTRICT LEVEL**

- Motivated and higher performing health workers and increased health facility functionality
- Improved health service delivery outcome indicators in implementing districts, e.g. ANC, institutional delivery, TB case finding, Childhood immunisation

**INPUTS**

- Process evaluation of the pilot district
- Context analysis of implementation districts (3- Mountain, Hill and Terai)
- Adapt and refine Health Worker Performance Model in an interactive workshop (informed by pilot and district context analysis)
- Train District Manager, District Supervisors, Health Workers and members of Health Facility Management Committee on HWPM
- Implement intervention (group monitoring, supportive supervision and feedback, improved performance appraisal system),
- Monitoring and evaluation - an ongoing process
- Methods used: Qualitative approaches (in-depth interviews, focus group discussion, ethnographic observations, and participatory observation techniques); and Quantitative approaches (baseline and endline data on health service delivery indicators using record review of district-level facility data, analysis of data through time, staff motivation will be measured before, during and after the intervention using a tool for assessing health worker motivation and satisfaction)

**Aim:** To assess the processes and effectiveness of a health workforce performance model (HWPM) in improving staff performance and health outcomes and its scalability in Nepal

## Expected outcomes

The overall outcome of this study will be a clear understanding of the most appropriate way to implement the health worker performance model (HWPM). In particular, the study will identify what works in different geographical, cultural, governance and health facility contexts, why and how the model works in these contexts. The strong links of the study team with national policy makers means that we also expect this understanding of how the model works to influence the HR policy and practice across Nepal. In the most effective decision making environments, all relevant parties – researchers, decision-makers and other stakeholders, including civil society actors, work together as interdependent allies in an environment of mutual trust and respect. This enables major decisions to be based on a solid foundation of evidence and benefit from a broad range of inputs. Throughout this study we intend to work with decision-makers to create this positive environment for change in HR systems in Nepal’s health sector.

The output of the study will be a detailed, documented understanding of the processes involved in the HWPM in Doti and then in the implementation of the model in three districts from different geographical and cultural regions, with different levels of performance and functionality of governance systems and in all levels of health facility. This detailed understanding will increase the knowledge available to policy makers and practitioners wishing to implement the HWPM in different context. To aid them to do this a HWPM implementation guide and training manual will be produced.

In order to develop this detailed understanding we propose conducting detailed case studies, at least 4 in each of the 3 districts, of the process of implementation of the HWPM over a 20 month period. These case studies will each be centred on a health facility, and in each district a health facility of each level (Hospital, PHCC, Health Post and Sub Health Post) will be included. This will give a detailed understanding of implementation issues at different levels of the health system and for different cadres of staff. The purposive sampling will ensure that perspectives from male and female staff of different castes and ethnicities captures any variations in how the HWPM can best work with all staff.

*Anticipated effectiveness outcomes:* increased motivation of health care workforce delivering quality health care services, Increased functionality of health facility and reduction in staff absenteeism*;* Improved health service delivery outcomes (i.e. maternal and child health)

In-depth documented context specific (district) *understanding* of the processes involved in the intervention from the perspectives of different stakeholders

*Anticipated effectiveness outcomes:*

- **Increased motivation of health care workforce delivering quality health care services:** motivation of health workforce will be measured before and after the intervention using a tool for assessing health worker satisfaction. Two tools will be piloted in Doti and an adapted tool appropriate for the Nepal context will be used to measure motivation before and after the intervention in three districts. ^1,2^

Staff motivation is seen here as a process outcome that ultimately influences health service utilisation outcomes, particularly maternal and child health. The two years of this study intervention may not be sufficient to see impacts on health outcomes, however these will still be collected as secondary outcomes in order to explore possible relationships. The qualitative data will also help to elucidate these linkages and motivation level at different stages of intervention.

- **Increased functionality of health facility and reduction in staff absenteeism:** functionality related outcome to be measured using facility records: service utilisation register (OPD), and staff attendance register. The proportion of official open days for which the clinic is open and the provider is in place before and after the intervention will be measured. Qualitative interviews with community people (including households nearby health facility), service users, and members of health facility management committee will be conducted to validate facility records.
- **Improved health service delivery outcomes:** all health service delivery outcomes measure proposed are based on data that is routinely available through the HMIS system. Measures will be assessed before the intervention is implemented and one year later. Qualitative interviews will be conducted to further understand what drives the success or otherwise of the intervention. The following health service delivery indicators will be measured: the proportion of deliveries conducted by a skilled birth attendant at home and in a government health facility (denominator is expected pregnancies DOHS); the proportion of women who have 4 ANC checkups (denominator is expected pregnancies DOHS); Childhood immunisations: the proportion of children who receive DPT1 who also receive DPT3 within 4 months after birth before and after the intervention.

**Dissemination/scalability:** These outcomes are strategic in supporting scalability, the primary outcomes provide a tested model with supportive documents to guide scale up and in depth understanding of the processes within. Monitoring the secondary outcomes will help further develop the evidence base. Dissemination of the study will be done in presence of officials of Department of Health Services and the Ministry.

## Study design/ methodology

It is a mixed methods intervention research with a focus on process evaluation using qualitative approaches. The effectiveness of the model will be compared using baseline and end-line data on health workers’ motivation and health service delivery outcome indicators. The processes followed during implementation and the influence of context will be explored in depth in 16 case studies covering all levels of facility in each of the districts.

##

## Procedures

This study will follow the following steps:

**Step 1:** documentation from the Doti pilot and analyse the context in 3 intervention districts (Obj 1 and Obj 2)

**Step 2:** develop a Health Worker Performance Model (Obj 3)

**Step 3:** test the model in three intervention districts and document lessons and the processes involved (Obj 3)

**Step 4:** finalise a tested, costed and improved HWPM and an implementation guide and training manuals (Obj 3)

The table below provides a summary of the methods that will be used to meet each objective. The procedures are outlined in greater detail under the steps identified below.

Table 4: Summary of Methods by Objective

| **Objective** | **Method** | **Analysis** | **Time Frame** |
| --- | --- | --- | --- |
| **1.** To identify lessons from Doti District pilot intervention on improving effectiveness and efficiency of health workers and develop an understanding of what worked, how and why; | Document /Record review to identify trends in key indicators.  In-depth interviews with purposively sampled key informants (HW, district staff and community perspectives - HFMC) to capture experiences through time from initiation of package to current day.  Pilot the health care worker motivation tools to determine which tool works best in the Nepal context and to make any changes to the translation of the tool for the Nepal context. | For routine data and motivation tool: descriptive statistics  For qualitative data: Framework Approach | Months 1 to 6  NB Motivation tools to be piloted by month 2 in order to inform tool used in contextual analysis. |
|  |  |  |  |
| **2.** To analyse the context in the 3 intervention districts (mountain, hill and plains) and ascertain implementation issues and strategies | 20 semi structured interviews (5 in each district) with purposively sampled key informants (HW and district staff) to provide diversity in terms of gender age, permanent/temporary contract and length of experience to identify current practices in monitoring and supervision and any other key HR issues.  Document /Record/facility data review to identify baseline in secondary outcomes and to identify any other HR initiatives.  To collect base line data using the motivation tool. | For qualitative data: Framework Approach  For routine data: descriptive statistics | Month 1 to 6 |
|  |  |  |  |
| 3. To develop, refine and test a HWPM model in 3 different districts, assess processes, effectiveness and scalability | Initial intervention design workshop.  Mid point national workshop for implementing districts to share experiences and lessons learned and to inform Nepal Health Sector Development Programme 3.  In-depth interviews, focus group discussions and ethnographic observations. Iterative adaptation of the intervention package where appropriate.  Reflective workshops for HW and district officials in each district on the successes and challenges of the intervention.  Collect and review health facility data and routine HMIS data through time  Follow up health workers from baseline and repeat collection of motivation questionnaire.  Assess basic costs of the model  Ongoing policy and influencing work, through engagement with MoHP working groups and structures on human resources and workforce performance. | Framework analysis of qualitative data (reflective workshops, IDIs and observational data) to identify barriers and facilitators.  For routine data: descriptive statistics  For Motivation questionnaire: descriptive and analytical statistics | Process evaluation data collection of implementation:  Month 7 to Month 20  Endline data collection and final analysis/report writing  Month 21 to 24 |
|  |  |  |  |

### **Study sites**

We will purposively select 3 study districts to capture the geographical and cultural diversity of Nepal. Given the issue raised in relation to recruitment and retention in the different geographical contexts of Nepal, understanding what makes the model work in these different settings is vital. In light of this, districts selected are from each of the 3 main ecological zones, Rasuwa (Mountain), Dang (Terai), Baitadi (Hill). Developing and understanding of how the intervention works in these different environments will facilitate scale up across the country.

Figure 3: Map of Nepal showing pilot and intervention districts

Table 5: Overview of health facility and HR in intervention districts

| **District** | **Region / ecology** | **Number of health facilities in each district** | | | | **Approximate number of Heath staffs/ facility** | | | | **Approximate total no of health staffs** |
| --- | --- | --- | --- | --- | --- | --- | --- | --- | --- | --- |
|  |  | **Public Hospitals** | **PHCCs** | **HPs** | **SHP** | **Public Hospital** | **PHCC** | **HP** | **SHP** |  |
| Rasuwa | Central/Mountain | 1 | 1 | 14 | 2 | 20 | 9 | 5 | 3 | 105 |
| Dang | Mid western/ Plain (Terai) | 2 | 3 | 15 | 21 | 20 | 9 | 5 | 3 | 205 |
| Baitadi | Far western/Hill | 1 | 2 | 15 | 50 | 20 | 9 | 5 | 3 | 263 |

### **Methods**

#### *Step 1: Documenting Doti and Context Analysis*

A mix of quantitative and qualitative methods will be used to identify the lessons learned in Doti and also to analyse the context and set the base line for the three intervention districts.

#### *Step 1.1: Lessons Learned in Doti*

In order to understand the context and how and why the model worked so well in Doti, the study will use a mixture of quantitative and qualitative methods.

- **Document Review**: Documents highlighting HR systems and developments over the time the model was first developed will be analysed in particular looking for systems changes and processes that may have been put in place and any reactions to these from any part of the health economy. Assess the number and cadre level of locally contracted and temporary staff, the transfer of staff, any absenteeism and functionality of the facilities.
- **Analysis of Routine Data**: Routine data will be assessed to look at changes in the health outcomes we have identified above and comparison with similar districts to see any possible improvements. At least 5 years of data will be assessed to determine any changes over the period of the intervention. This will also help us to test the feasibility of collecting this data and adapt any tools or processes to ensure that data collection during the main implementation of the model is effective.
- **Pilot the health worker motivation** tools with a range of health workers.^1,2^ As neither of these tools have yet been used in Nepal, we are keen to pilot both the tools and identify which one works best in the Nepal context. It may be that a combination of the two tools is developed that best covers all aspects of staff motivation.

Doti has approximately 217 staff spread across 2 public hospitals, 2 primary health care centres, 10 health posts and 39 sub health posts. As the use of the tool in Doti is only a pilot, a formal sample size calculation is not required, instead 30% of the total number of health workers (217) in the district will be sampled (65). The tools will be used with staff of different levels and genders to understand any issues in its implementation. The following participants will be recruited to fill the motivation questionnaires:

#### *Doti: Piloting of Motivation Tools*

Table 6: Tool piloting in Doti

| **Health Facility/administration level** | **Total number of facilities and approximate number of staff in Doti** | **Required Staff roles at each level of facility (NB in practice posts may not be filled)** | **To be sampled**  **(Ensuring gender balance)** |
| --- | --- | --- | --- |
| DHOs Office | 1 District Health Office | District Public Health Office and administrative officer for HR and District Supervisors for Programmes | 3 male and 3 female to include DPHO, Admin Officer responsible for HR, 4 district supervisors |
| Public Hospital | 2 Hospitals = approx 40 staff |  | 10 x 1 hospital = 10  Gender and staff level balance |
| Primary Health Care Centre | 2 x PHC = 10 staff | 1 Medical Officer, 1 Health Assistant,  1 staff nurse  3 Auxiliary Nurse Midwife  3 auxiliary health workers | 5 x1 PHC = 5 |
| Health Post | 10 x HPs = 50 staff | 1 Health Assistant,  2 Auxiliary Health Workers  2 Auxiliary Nurse Midwives | 5 X 4HPs = 20 |
| Sub Health Post | 39 x SHPs = 117 staff | 2 Auxiliary Health Workers  1 Auxiliary Nurse Midwives | 3 x 8 SHPs = 24 |
|  |  |  | Total: 65 |

#### *Doti: Qualitative interviews with health facility staff:*

The Motivation Tool will also be used to purposively sample health workers for qualitative interviews. Once the questionnaire has been completed the interviewer will identify whether the participant is highly, poorly or moderately motivated. Based on this, the interviewer will then ask if the participant is willing to continue discussing their experiences of the HR model being used in Doti though participation in a qualitative interview. This will allow reflection on why the model may be more effective for some health workers and not others. It will also allow greater reflection on the validity of the motivation tools. For example, during the qualitative interview it may become apparent that the participant is not actually motivated even though they appeared to be from their score from the motivation tools. This will help the team reflect on which of the two motivation tools appears to be the most sensitive.

In this way, it is estimated that ten in-depth qualitative interviews will be conducted. Interviews are felt to be an appropriate method as such individual experience of the working environment are confidential and participants are unlikely to share such information in a group setting. In addition to the results of the motivation tool, staff will be purposively sampled based on:

- Working in the district for the last 5 years (in order to have sufficient institutional memory to reflect on the processes and experiences when the model was first implemented)
- Gender balance – an equal number of men and women
- Different cadres of staff
- Types of contract – permanent and temporary

*Purpose of Interviews with Staff*

- To hear their experiences of the HR model – what works well and what does not, if and how it has helped them to improve their performance and level of motivation, how it works in practice
- To understand their level of motivation better in order to reflect on the validity of the Motivation tool.

#### *Doti: Qualitative Interviews with Health Facility Management Committees (HFMC)*

As representatives of their communities, and those able to observe the day to day functioning of the health facilities, members of HFMCs have a valuable perspective on their health facilities. In light of this, qualitative interviews will also be conducted with HFMC members in the sample of the facilities visited. Individual interviews are felt to be appropriate as there may be factors that are individually sensitive, for example negative experiences with health facility staff, or gender based discrimination on the committee. Approximately ten interviews will be held with HFMC members who will be purposively sampled based on:

- Gender
- Ethnicity/caste
- Level of health facility to which their HFMC is attached.

*Purpose of Interviews with HFMC members*

- To hear their experiences of the HR model – how it works in practice
- Whether they have noted any changes in the facility or staff over the implementation period of the model.

*Doti: Analysis of Data*

A data base will be set up in CSPro for data entry of the motivation tool data. This data will be analysed as pilot data to inform the final tool to be used, and also to guide the development of the data entry database and the analysis plan. The qualitative interviews will be recorded, transcribed and then analysed using the Framework Approach.^12^

*Output from Doti:* A paper will be written detailing the intervention used in Doti, and identifying the potential for the intervention to work in different settings and for different types of staff. The analysis of the Doti experience will help to further develop the conceptual framework to explain the processes at work which influence the effectiveness of the intervention in different contexts. These ‘theories’ can then be further tested in the main study in three districts.

The experiences from Doti will also be fed into the finalisation of the design of the intervention.

#### Step 1.2: Contextual Analysis in three districts

In order to build up a comprehensive picture of the three intervention districts a mixture of routine data, quantitative and qualitative data will be collected.

*Document Review:* Policies relating to HR, supervision reports, minutes of meetings relating to HR, review of records of health workers, review of registers in health facilities

To identify:

- Posts held in each health facility, recruitment gaps, long term leave, assess the number and cadre level of locally contracted and temporary staff.
- Current HR practices, particularly in terms of supervision, recruitment, career development
- Number /type of staff on permanent/temporary contracts
- Other HR initiative in places
- Staff absenteeism (from health facility records to be verified through qualitative interviews)
- Health facility opening hours (official hours, and to be verified through Focus Group Discussions (FGDs) with communities)
- Health outcome indicators from district Health Management Information System (HMIS) data and facility registers to set base line.

*Semi-structured Interviews (maximum 42) with District Officers:*

Participants:

- At District Health Office approximately 4 interviews: District Public Health Officer (DPHO), Administrative officer responsible for HR and district supervisors
- At Health Facility Level approximately 10 interviews balancing male and female: HFMC members (one from PHCC level, HP and SHP level); health workers of different levels, gender and caste from all cadres (Medical Officers, ANMs, AHW, Health Assistants), female community health volunteers.

Approximately 14 interviews in each district will result in 42 overall. This is a large number of qualitative interviews and saturation may well be reached before all 42 have been completed.

Analysis of interviews will be conducted in tandem with data collection, so the themes emerging from the early interviews can inform the development of the topic guides and issues discussed in later interviews. For example a particular gender issue may emerge and the team decide to interview further female staff of that cadre to identify how common the particular experience is among female staff.

Purpose/key topics for semi structured interviews with district level and facility staff:

To understand:

- what motivates staff of different levels and how they feel about their work
- current supervision practices,
- what has helped to change motivation and performance in the past
- recruitment practices and reasons why some posts are hard to fill
- perception of the extent of and reasons for absenteeism, local contracting practices,
- career progression and any particular constraints facing female staff or staff of disadvantaged caste groups
- Understand engagement of communities and accountability to communities

*Focus groups discussion (6) with community members:*

Participants: community members, male and female groups separately, caste/ethnicity separately. Two focus groups will be held in each district, so six will be conducted in all. Focus groups have been selected as an appropriate method as, particularly where participants share a common experience or characteristic, they help participants to talk openly and provide greater depth of discussion on the facility and it’s functioning (Denzin and Lincoln, 2001)

Purpose/key topics for focus group discussions:

To understand:

- community perceptions of the health facilities of different levels in their area e.g. opening hours, availability of health workers, friendliness of HWs, quality of service, infrastructure and equipment at health facilities, availability of essential drugs and free services, changes over time for all of the above.
- The accountability of health facilities to their community, particularly the role of the HFMC, functionality of the HFMC, its representation of the community (including women and disadvantage groups).

Purposive Sampling of Community Members

Community members will be recruited from several sources in order to provide different perspective, particularly based on gender, caste and ethnicity. In each district three FGDs will be held:

- Local leaders: E.g.VDC chair, school teachers, FCHVs to provide a broad level perspective of the functionality of the health facility and the performance, availability of the health workers.
- Women groups: women often make up the majority of users of primary health care facilities, mainly due to reproductive health needs and as primary carers of small children. Women are more likely to speak freely in focus groups where men are not present.
- Patients: this focus group will be made up of patients found visiting the health facilities when the researchers visit the health facility, they will purposively sample patients from disadvantaged caste groups such as Dalit and Janajati to ensure that their perspectives are heard.

*Motivation Questionnaire:* Once the two motivation questionnaires have been piloted in Doti, the research team will agree on the tool to be used and the translation will be finalised. This decision will be based on:

- The sensitivity of the tool in relation to the qualitative interview data collected from participants who also filled in the 2 motivation tools
- The interviewers reflections on how easily the participant understood the questions in each tool, whether they had to be repeated etc.
- The length of time and ease of completion of the questionnaire.

This final tool will then be used in the context analysis to collect base line data on the level of staff motivation. A sample of 280 health workers spread across the facilities in the three districts will be required.

*Documentation Review at Regional and Central level*

This will include reviewing and synthesising available data on policy and practice: HR Strategic Plan 2003 to 17; National Health Policy (1991); Second Long-term Health Plan 1997-2017; NHSP-2; HR Strategy for achieving MDGs – including deployment and retention of HR, supervision, barriers and facilitators.

#### Step 2: Developing and Introducing the Intervention

Based on the lessons learnt from the Doti work, the context analysis and from HRM evidence, the intervention will be finalised. Section 2.3 describes the basis for the proposed intervention. An initial intervention design workshop will be held with the full research team and key stakeholders from each district.

Exactly how the elements of the HR model are operationalised in each district will be developed in close collaboration with the District Health Office (DHO). In order to prepare the health facilities for this new way of working workshops to introduce the new approach to monitoring and supervision and the development of job aids will draw on the techniques used in Appreciative Inquiry. These approaches help health workers to appreciate the benefit that they can provide to their communities and identify the assets they have as individuals and within the health system to provide quality care. A national workshop for the three implementing districts will be held six months into implementation to share experiences and lessons learned and to inform Nepal Health Sector Development Programme 3. Both the initial workshop and this mid-term workshop will provide valuable data for the process evaluation. As such, a detailed record of both workshops will be kept and analysed as part of the qualitative analysis. The mid-term workshop in particular, may highlight process issues that can be followed up through individual interviews and observation by the research team.

#### Step 3: test the model in three intervention districts and document lessons and the processes involved

The process of implementing the intervention will be observed and documented over a 14-month period. Case studies will be developed for at least one level of facility (i.e. hospital, PHC, HP and SHP) in each district.^13^ In Dang and Baitadi where there are more HPs and SHPs, two of these may be taken. Ultimately this will lead to sixteen case studies. The health facilities for the case studies will be selected to give a variety of performance levels. This selection process will be done based on MOHP performance indicators and the advice of the DPHO. The case studies will build up a picture of the wider context as well as showing any changes over time to show what elements of the HR model work well, for whom and in what contexts.

The methods within these case studies are as follows:

*Observation of Group Monitoring***:** Observation of the group monitoring and community assessment processes will be an important element in building up the case studies. For some facilities, the research team should be able to observe two group monitoring community assessments visits as these are planned to be held annually. This will allow the team to build up a picture of changes both within the facilities, the staff themselves and the involvement of the community and their perceptions of the health facilities.

*Purpose of Observations:*

- To identify the extent of community involvement, who in the community voices their opinions (giving special attention to age, gender, caste and ethnicity) and how far community perspectives are reflected in the health facility action plans
- To reflect on the level of engagement of facility staff with the monitoring process, whether this is seen as supportive or as interference, why these different perspectives arise and how this influences the actions for improvement identified.
- To document and explain all the outputs of the group monitoring and community assessments.

*In-depth interviews with health workers* of various cadres and to give gender and caste/ethnicity balance will be held.

Purpose of the interviews with health workers:

To explore:

- Experiences of supervision and monitoring – how regular and who by, how supportive, has it changed in light of the intervention, how has it influenced their work and motivation.
- Perceptions of the group monitoring and community assessment – how do they feel about this process, how does it affect their motivation, work pattern, sense of purpose.
- Opinions on new job aids – have they had any impact on the individual and their working practices
- Any experiences or issues arising in the workplace relation to gender, caste, ethnicity, type of contract or other individual characteristic.

***In-depth interviews with HFMC members*** particularly women and those of disadvantaged caste/ethnicity groups. If possible the researchers will also observe HFMC meetings in the case study heath facilities in order to triangulate with the findings of the interviews.

Purpose of the interviews with HFMC members:

- To assess the functionality and role of the HFMC
- To explore how far the HFMC is in touch with and responsive to community needs
- To explore their perceptions of any changes in the health workers and functioning of the facility in light of the HR model implementation.

*Focus groups with community members* delineated by gender, case and ethnicity

Purpose of the community FGDs:

- To hear their views on the HFMC, how far it represents their health needs
- To explore the knowledge of and experience of the community assessments done during group monitoring
- To explore their perceptions of any changes in the health workers and functioning of the facility since the HR model implementation.
- To identify any particular constraints experienced by women and disadvantaged groups in the community.

*In-depth interviews with District Officials,* in particular the DPHO, administrative officer responsible for HR and district supervisors. Who is interviewed will also be influenced by issues coming from facility level.

Purpose of interviews with District officials:

- To understand how well the model is being implemented, what is working well and what is not, challenges and successes.
- To identify and discuss wider context issues, such as central policy changes, resource limitations/changes, other HR interventions.
- To triangulate information from facility level.

*Reflective workshops* for Health Workers and district officials in each district will be held once a year in order to reflect on the successes and challenges of the intervention.

*Routine data collection:* The team will also collect and review health facility data and routine HMIS data through time.

*Collection of cost information:* The research team will document the costs of the intervention in particular recording in each district:

- Costs of group monitoring and community assessment of all health facilities
- Approximation of time spent on supervision, this data will be gathered in the case study facilities and the amount of time checked at district level for consistency with other non-case study facilities.
- Costs of district level workshop and development of job aids.

Based on this evaluation of the on-going process of the intervention, the team will keep in close contact with the DHO to allow iterative adaptation of the intervention package where appropriate.

#### Step 4: Finalise a tested, costed and improved HWPM and an implementation guide and training manuals

*End line Motivation tool:* The same health facilities visited during the baseline data collection will be visited again during the end line and the sample size of 280 will be recruited. While following up individuals cannot be achieved as this might undermine validity as health workers would be concerned about confidentiality, this approach will provide and strong indication of changes in health worker motivation over the course of the intervention.

*End line Collection of routine health service outcomes data* for indicators i.e. institutional delivery, delivery by Skilled Birth Attendant, 4 ANC visits, TB case finding and treatment outcome.

*Conduct Final Analysis*: The qualitative data will be analysed throughout the study in order to inform the emergence of themes, the further sampling of participants and interview guides to be used. In this last phase the case studies will be written up and themes that emerge across the case studies will be identified, in particular the factors influencing the success, or not, of the implementation of the model and how the different contextual factors influence this. The quantitative and qualitative data will be triangulated to explore, for example any anomalies where by health facilities whose staff appear very motivated (from the Motivation tool) but have poor indicators from the routine data. The qualitative data will help illuminate and explain anomalies and bring understanding to why the intervention may work or not is certain circumstances.

*Assess basic costs of the model:* the costs of the intervention will be collated and analysed. Incremental cost effectiveness will be calculated to show the cost per change on the motivation tool.

*Ongoing policy and influencing work:* throughout the intervention the team will engage with MoHP working groups and structures on human resources and workforce performance. In particular, the team will ensure that lessons learnt from this model are fed into the Implementation plans following the HR Strategic plan and the design of NHSSP 3.

### **Sampling**

Measuring Change in Motivation: Sample Size Calculation

The piloting of the motivation tools will provide more appropriate Nepal-specific information such as between individuals standard deviation (s.d.). However, for the purposes of planning the Mbindyo et al’s scale has been used to estimate the required sample size in order to detect a psychologically/clinically significant difference before the intervention and 20 months later.^1^ Mbindyo et al found a between individuals s.d. of 11 units.^44^ The mid-point of the Mbindyo et al scale is 30.- Assuming a 10% change (i.e. 3 points) is of clinical/ psychological significance then across the three districts, a random sample of 280 health workers will be invited to complete the motivation questionnaire at baseline; a different random sample of the same size will be selected post-intervention.  Assuming around a 10% refusal rate, this should provide 250 analysable questionnaires at both assessment times.  The mid-point of the motivation scale is 30 points; assuming a standard deviation of 11 points, this sample size will provide 86% power to detect a mean change in mean motivation score from baseline to post-intervention of 10% (3 points), with an alpha level of 5%.  The detectable change in mean score, with 90% power, at each of the participating districts is: Rasuwa 16% (4.7 points); Dang 18% (5.3 points); Baitadi 25% (7.5 points).

Table 7: Sample size of health workers in three districts

| **District** | **Region/ecology** | **Required Sample**  **Size** |
| --- | --- | --- |
| Rasuwa | Central/Mountain | 51 |
| Dang | Mid western/Plain (Terai) | 100 |
| Baitadi | Far western/Hill | 129 |
|  | Total | 280 |

Sampling of Health Facilities

In order to provide a spread of the required sample size across the different health facility levels, half of the total number of each level of health facility will be sampled. The required number of sampled health facilities will be recruited to give a spread based on high, low and moderate performance. This sampling process will be done in close collaboration with the DPHOs office in each district.

From these facilities the required sample of health workers will be recruited. Recruitment will be monitored to ensure that there is the required representation of the different levels of health facilities, health worker cadre and gender balance. If any of these groups appear to be disproportionately being recruited then remedial action will be taken.

Table 8: Health facilities to be recruited in the study

|  | **Hospital** | **PHCC** | **HP** | **SHP** | **Estimation of available health workers for recruitment** | **Required Sample**  **Size** |
| --- | --- | --- | --- | --- | --- | --- |
| Rasuwa | 1 | 1 | 7 | 1 | 67 | 51 |
| Dang | 1 | 2 | 7 | 10 | 103 | 100 |
| Baitadi | 1 | 2 | 7 | 25 | 148 | 129 |
|  |  |  |  |  | 318 | 280 |

Qualitative Sampling

Health workers will be purposively sampled to provide age, gender, caste/ethnicity balance, and also an even representation of different cadres of staff. The approximate numbers are indicated in the section above, however these may change in order for data saturation to be reached on particular issues. There will also be an element of ‘theoretical sampling’^14^, whereby, if explanations – or theories – begin to emerge that highlight why the interventions may be working for some staff and not others in different contexts, then the sampling strategy for qualitative interviews will be adapted to explore these issues further with the relevant staff. This will give deeper insights into why the intervention may or may not work for different staff in different contexts.

Community members will be purposively sampled for the focus groups which are part of the context analysis. As described above, this will be based on gender, class, caste and ethnicity. There has been a policy move within Nepal to ensure the HFMCs have representatives from disadvantaged caste and ethnic groups, particularly Dalit and Janajati, and also that there is gender balance. In order to explore how far this diversity is achieved in practice and how to work with HFMC’s to improve representation of disadvantaged groups.

### **Analysis**

*Motivation Tool:* All quantitative data will be double entered into CsPRO. Checks will be run to identify missing data and any anomalies. Data will be exported to STATA to carry out the quantitative analysis. The characteristics of health workers and their places of work will be presented for each district to identify any significant differences that may lead to bias. The difference in mean scores from the motivation of HW tool before the intervention and after will be analysed using ANOVA to identify any significant differences (shown with 95% CIs and p values). Regression analysis will be used to identify the influence of various variables on the main outcome of staff motivation. For example, difference due to gender, age, level of health worker, place of work (hospital, PHCC, HP, SHP). Factors such as other HR initiatives or health systems changes will also be recorded and included in the analysis as they potentially be confounders.

*Routine Health Service Data:* Frequency tables with proportions will be used to compare secondary outcomes across districts. Where possible trend data from health sector annual reports over the last 5 years will be used to avoid over interpretation of data peaks.

*Qualitative data:* Ritchie and Spencer’s ‘framework’ approach will be used to analyse the qualitative data.^12^ The advantage of this structured approach is that the thematic framework can be closely based on the research objectives but also allows for other themes to emerge from the data. The transparent structured approach supports collaborative analysis. There are 5 key stages within framework approach:

1. Familiarisation: Here the analyst reads through transcripts and notes pulling out key ideas and starting to identify recurrent themes. This process will be done by two HERD researchers independently.
2. Identifying a thematic framework: In this step of the process a framework is constructed based on issues from the original research aims, emergent issues from the participants and the recurrent themes identified during the familiarisation process. At this point the cross-cutting themes of motivation, gender, management styles, other health system issues within the framework to see how perceptions and issues differ across these groups. The 2 LSTM (ST and JR) consultants will work with HERD researchers to develop the framework.
3. Indexing: Here the thematic framework is applied to the data.
4. Charting: this process involves lifting data from the original context and placing it within the appropriate thematic categories within the framework.
5. Mapping and interpretation: The final stage in the process is to interpret and map the range, differences and similarities within the data presented in the charts.

### **Strategies for ongoing engagement of key stakeholders**

This study has been designed to support ongoing engagement of key stakeholders and scalability. The leading role of the DPHO for Kathmandu District as a key decision maker within the government health sector and the experience of HERD in engaging with MoHP to influence decisions mean that scaling up the approach across the country is viable. The HR Strategy is still in its infancy, evidence of how to practically address the objectives of the strategy is urgently needed in order for the strategy to have an impact nationally. This study, and its strong connections with District health structures and MoHP has the potential to have an important and timely impact on policy; and will feed into the design of the 3rd National Health Sector Programme.

There will be continual engagement with MoHP and key practitioners throughout the life of the project through the following strategies:

- Steering Group meetings (as detailed in section 2.8.1) will be held every 3 months and will include active participation of key decision makers within the government health sector
- Participation of key policy makers and practitioners within the methodological design of the implementation research project (for example in the initial intervention design workshop, the midpoint national workshop and the reflective and interactive workshops)
- Participation and sharing of project process and outcomes within regional and national health reviews, which are attended by all DHOs, regional directorate and senior officials from the ministry of health
- Participation in Joint Annual Review (JAR) - a high level fora which brings together the ministry and external development partners
- The research team constitutes academia, local researchers, policy makers and implementers which help ensuring strategic engagement of relevant stakeholders
- Proposal focus fits within national priority area, is of interest to key stakeholders and will feed into ongoing discussion and debates in NHSP 2 and development of NHSP 3
- Proposal builds directly on the success and innovation taken forward by a DHO, and there is a core interest group of DHOs (implementation districts) who will be engaged throughout the process
- The proposal has a number of approaches designed to foster engagement of stakeholders throughout i.e national, regional and district level workshops, meetings, dissemination meetings

## Data quality assurance/ management

*Quantitative Data Collection:* The motivation questionnaire will be conducted by enumerators trained to collect the data correctly. A supervisor will check all questionnaires for quality. As the questionnaire is a straightforward list of 10 questions on a scale of 1 to 5, it is not envisaged to cause major data collection issues. However, questionnaires will be checked throughout the process of data collection to ensure that they are complete and filled correctly. All quantitative data will be double entered into CsPRO. Checks will be run to identify missing data and any anomalies. Data will be exported to STATA to carry out the quantitative analysis. All questionnaires will have a unique identifier which will not only ensure complete anonymisation of the data, but will also enable good data management and the ability to go back to original questionnaires to check any inconsistencies or incomplete data.

*Routine Data***:** Routine data will be collected from HMIS reports at district level and verified at the sampled health facilities. This will give an indication of the completeness and accuracy of the data. Any anomalies can be raised with the health facilities.

*Qualitative Data:* HERD’s researchers are well trained and experienced in the use of qualitative methods, including interviews, focus group discussions and observation. Interviews and focus group discussions will be recorded using digital recorders and written up as soon as soon they take place wherever possible. The researchers will also keep a reflective diary which can be used to inform the analysis and to understand why certain methodological decisions have been taken. The use of several members of the team to develop the coding framework and reflect on the analysis will provide inter-rater reliability of the qualitative findings.

#

# Gender considerations

Health staff within the districts are both female and male and will benefit from the HWSP. We will understand the specific concerns of different cadres of female and male staff during the Doti evaluation and the contextual analysis of the intervention districts and ensure the refined HWSP responds to these needs. For example, nurses in Nepal are all female and often find it difficult to remain in remote areas and we address these concerns where appropriate in the HWSP.^7^ Each district also has 700 female community health volunteers, who should benefit from the intervention through enhanced supervision processes.

There is a clear need to improve health outcomes in Nepal, particularly in areas of sexual, reproductive and maternal health. Given the Doti experience we anticipate that the HWPM may impact positively on the ability of poor women, men, boys and girls to access quality health services.

The HWSP includes a process of developing and refining job aides, and this will include a focus on processes, for example ensuring greater support to women and particularly marginalised communities. As part of the process evaluation we will track service delivery indicators by gender, caste and ethnicity, and identify gaps and respond accordingly.

We will do our utmost to ensure that both female and male staff benefit from the HWSP and are able to participate in research processes to evaluate its impact. As detailed in our methods within the health sector and at the community level we will capture male and female perspectives and also ensure representation by age, class, caste and ethnicity. The team will do their utmost to ensure that interviews and data collection is done at a time and a place that is convenient for participants.

The core team has 4 men and 3 women. There are 4 men (Baral, Shrestha, Subedi and Martineau) and 3 women (Elsey, Theobald and Raven). The core team and their roles and responsibilities are outlined in section 2.8.1. All team members are committed to promoting gender equity throughout the research process.

#

# Ethical issues

The study team will work closely with District Authorities and community leaders to ensure that all stakeholders are well informed of the study and its purpose. The study will be reviewed by Ethical Review Committee of WHO, it will also seek ethical approval from the Liverpool School of Tropical Medicine and from Nepal Health Research Council (NHRC).

*Potential Issues:* health workers may feel unable to refuse participation or able to speak freely for fear of reprisals from senior managers. To address these issues during recruitment research staff will explain clearly that all data provided whether by questionnaire (motivation tool) or during qualitative interviews will remain anonymous. We will be careful in the characterisations used to locate quotations, for example not naming Districts directly (referring to Hill district or Terai District, rather than by name) in order to maintain confidentiality during reporting and dissemination.

All participants, whether they are health workers, district officials or community members will be given sufficient information about the study and be provided with opportunity to ask any questions and think over participation on their own before giving consent.

*Recruitment for Interviews with staff and HFMC members:* The research team will visit each of the randomly selected health facilities (as above) and present to all staff and to HFMC members: the study, its purpose and inclusion criteria for both the questionnaire and the qualitative elements. They will then leave information sheets and consent for all staff to read, allowing at least 24 hrs to consider their participation or not. They will make a time with the health facility to return to conduct the motivation questionnaire and qualitative interviews. At these start of these interviews the participants will be encouraged to ask any questions about the study. If they are still willing to participate, they will then fill in their consent form.

The researcher will be well trained to explain the study and the HWs right not to be involved should they wish so or leave the interview at any point of time. Qualitative interviews will be conducted at a private location away from the health facility to ensure confidentiality.

*Recruitment of community members:* If community members from the groups identified above are potentially interested in the study, the researchers will talk through the information sheet and consent process. If the potential participant is still interested in being part of the focus group, the researchers will let them know the time and the venue. At the start of the focus group the researchers will again go through the information sheet and take informed consent from the participants, including consent to record the FGD.

*Maintaining Confidentiality and Storage of Data:* All transcripts and questionnaires will be anonymised. The identity of the participants will not be known to others apart from the research team. The researcher will write the unique identifier on the consent form, once consent has been given and this unique identifier will then be used on all collected data. The consent forms will be kept in a locked drawer in HERD’s office which is locked and has a security guard. All data and analysis on HERD’s computers will be under a password protected system.

**References**

1. Mbindyo PM, Blaauw D, Gilson L, English M. Developing a tool to measure health worker motivation in district hospitals in Kenya. *Human Resour Health* 2009; **7:** 40.
2. Spector, P. Job Satisfaction Survey. Tampa: University of South Florida, 1994.
3. WHO. World Health Report. Geneva: World Health Organisation, 2006.
4. RTI International. Assessing Implementation of Nepal’s Free Health Care Policy: Third Trimester Health Facility Survey Report. North Carolina: RTI International, 2009.
5. RTI International. Human Resource Strategy Options for Safe Delivery. North Carolina: RTI International, 2009.
6. Ministry of Health and Population, RTI. Assessment of Health System Performance in Nepal HSRSP Report No. 2.20-5-10. Kathmandu: Ministry of health and Population, 2010.
7. Martineau T, Subedi HN. Human Resources for Health: Human Resource Capacity Assessment.  <http://www.nhssp.org.np/human_resources/Human%20Resources%20Capacity%20Assessment.pdf>. (Accessed 5 January 2013)
8. Thomas, D. and Subedi, HN (2010) Gender Equality and Social Inclusion Capacity Assessment and TA Design: An assessment of capacity building for health systems strengthening and the delivery of the NHSP 2 results framework. Kathmandu: Ministry of Health and Population, 2010.
9. Lehmann U, Dieleman M, Martineau T. Staffing remote rural areas in middle- and low-income countries: A literature review of attraction and retention. *BMC Health Serv Res,* 2008; **8:** 19.
10. Buykx P, Humphreys J, Wakerman J, Pashen D. (2010). Systematic review of effective retention incentives for health workers in rural and remote areas: towards evidence-based policy. Aust J Rural Health 2010; 8: 102–9.
11. Buchan J. What difference does ("good") HRM make? *Human Resour Health,* 2004; **2:** 6.
12. Ritchie J, Spencer L, O’Connor W. Carrying out qualitative analysis. In: Ritchie J, Lewis J, eds. *Qualitative Research Practice: A guide for social science students and researchers.* London: Sage Publications, 2003: 219-262.
13. Yin, R. Case Study Research: Design and Methods. 4^th^ Edition. Sage Publications, New York, 2009.
14. Glaser B, Straus A. The Discovery of Grounded Theory: Strategies for Qualitative Research. Sage Publications, New York, 2009.
