# Supplementary material for: What adaptation to research is needed following crises: a comparative, qualitative study of the health workforce in Sierra Leone and Nepal
Source: Health Res Policy Syst. 2018 Feb 7;16:6. doi: 10.1186/s12961-018-0285-1 (PMC5804047; doi:10.1186/s12961-018-0285-1)
Supplement: Supplementary file 4 — Nepal adapted study protocol. (DOCX 43 kb) [file 12961_2018_285_MOESM4_ESM.docx]

**Additional File 4: Nepal adapted study protocol**

On 25th April 2015, a massive earthquake measuring 7.8 in the Richter scale was experienced in Nepal. Continued aftershocks followed throughout Nepal till date. Fourteen districts were heavily affected by the earthquake, while many other districts are also reported to be affected. The heavily affected districts are Kathmandu, Sindhupalchowk, Nuwakot, Dhading, Gorkha, Rasuwa, Kavre, Bhaktapur, Lalitpur, Dolakha, Makwanpur, Ramechhap, Okhaldhunga and Sindhuli). Unfortunately, *Rasuwa is one of the HRH research implementation district.* Total 1003 public health facilities were damaged in all over the country. Among them, 439 were completely destroyed and 564 damaged partially. Total 784,484 households were damaged by the earthquake. Out of them 505,577 were completely damaged and 278,907 are partially damaged in all over the country. Likewise, total of 8,712 people died and 22,220 injured. Out of those who died, total 8 were health workers. Out of total injured 68 were health workers and 2 Health Workers are still missing. Similarly, total 10 FCHVs died and 7 were injured.

**Effect on the project district**

Among three research implementation districts (Rasuwa, Baitadi and Dang), Rasuwa is heavily affected from the earthquake with around 75% (15 out of 20) health facilities damaged, two health workers died, nine were injured and two health workers are missing. Similarly, 9,450 houses are damaged (7,040 fully and 2,410 partially) and Kathmandu Rasuwa highway and many other internal trails/roads are damaged heavily, increasing probability of landslides. As compared to Rasuwa, there is significantly less effect in Dang and Baitadi district. So, implementation of the PBMS model will be continued in Baitadi and Dang district, whereas it is difficult in Rasuwa.

Considering the need of immediate respond to the above circumstances, Steering Committee Meeting was held on June 25, 2015. The meeting suggested that, “since many of the health facilities are damaged and delivery of the basic health care service is heavily affected, full implementation of Health Workers Performance Model in Rasuwa is highly unlikely to be implemented. However, the selected tools of the model can be implemented in the limited damaged health facilities where service delivery function has been resumed and lessons learned in management of health workers performance in the aftermath of the earthquake. Similarly, the project will document the processes and draw lessons learned in the changed context (after earthquake) with a greater focus on health workers motivation and readiness to delivery basic health services in Rasuwa district. Meeting however suggested, continuing pilot research implementation in Baitadi and Dang as per the original plan.

**Capturing HW experience in Rasuwa**

Health Workers have remained in the health facility delivering services throughout the earthquake with no opportunity to go back to their families and recuperate. An important concern to address is to devise appropriate strategies to make health services more resilient to withstand these shocks as well as valuing, nurturing and supporting health workers in the given context. It is also important to understand the key challenges and enablers affecting the work performance of the health workers during emergency situations.

It is also important to explore the implementation of PBMS to date in Rasuwa. Most of the health facilities completed PBMS orientation, developed benchmarks and prepared facility level work plan by February 2015. Implementation was only possible for March and part of April, before the earthquake occurred All this information was lost in most of the health facilities except 3 or 4 less affected ones. Even in less affected health facilities, the situation became unmanageable as regular service delivery was interrupted for almost two months. Full implementation of the PBMS is unlikely to be feasible. Only selected tools can be used based on the local situation and experiences and past processes can be documented in these facilities. Comparison among health facilities is less feasible as the nature of service delivery varies among the health facilities.

**Study objectives**

The objectives of the study in Rasuwa:

- To explore health workers’ /managers’ perception and experiences of the implementation of PBMS to date;
- To describe the changes in service delivery and working environments after Earthquake in Rasuwa;
- To explore factors that influence health workers’ provision of health services before and after earthquake.

**Methods**

In order to document these experiences in an effective and efficient manner, qualitative methods will be used: observations of health facilities; semi structured interviews with health workers; semi structured interviews with managers; and semi-structured interviews with health facility management committee members. See table 1 for the methods, participants and areas to cover in the interviews and observations. Persons involved in this study will require considerable skill in qualitative research along with some skills on psychosocial counselling.

**Table 1: Methods, participants and areas to be covered in method**

| **SN** | **Method**  **(Tool)** | **Participants** | **Areas to cover in interviews and observations** |
| --- | --- | --- | --- |
| 1. | Observation (Checklist) | Selected health facilities | - Current working environment – whether it is encouraging for the health workers to perform in difficult circumstances; whether the health facilities are safe enough to seek health services |
| 2. | Semi-structured Interview  (topic guides) | Health workers | - Perceptions and experiences of PBMS implementation - Perceptions and experiences of post earthquake service delivery situation and their effort, willingness, motivation and accountability - Factors that influence provision of services - Recommendations and ideas to support them/help them cope with what they have been through |
|  |  | Health managers | - Perceptions and experiences of PBMS implementation - Perceptions and experiences of health service management, service delivery & role of service providers in post-earthquake situation - Factors that influence provision of service - Recommendations and ideas to support health facilities / workers / managers cope with what they have been through |
|  |  | Health facility management committee members | - Perceptions and experiences of PBMS implementation - Challenges in service delivery post-earthquake - Recommendations and ideas to support health facility / health workers cope with what they have been through |

**Sampling for facilities and participants**

*Facilities:*

Five facilities in Rasuwa district will be selected using the following criteria: received support in implementation of PBMS and monitoring from research team; did not receive support from research team, but started implementation of PBMS; different levels of facility; and degree of damage caused by earthquake. The proposed facilities are shown in table 2.

**Table 2: Proposed facilities selected for study**

|  | **Name of facility** | **Received support from research team** | **Level of facility** | **Damage by earthquake** |
| --- | --- | --- | --- | --- |
| 1 | Jibjibe PHC, | Yes | PHC | Less damaged |
| 2 | Syafrubesi HP | Yes | HP | Very damaged |
| 3 | Parchang HP | Yes | HP | Very damaged |
| 4 | Laharepauwa HP | Yes | HP | Less damaged |
| 5 | Gatland HP | No | HP | Very damaged |

*Participants:*

- Health workers: approximately 2 health workers from each facility who participated in the PBMS training at facility level will be interviewed; we will try to ensure a range of cadres i.e senior ANMs , ANMs, senior AHWs and AHWs, and a mix of male and female health workers. There will be a total of eight interviews with health workers.
- Health managers: we will select the DHO, PBMS focal person, PHC in charge, and HP in-charge, as they have all been involved in the PBMS programme, are members of the District Performance Evaluation Committee, and are supervisors of health staff. There will be a total of 4 interviews with managers.
- Health Facility Management Committee (HFMC) Members: we will select one member, who has been involved in the PBMS programme, from two HFMCs. There will be a total of 2 interviews with HFMC members.

Table 3 shows the numbers of interviews and observations to be conducted at selected facilities and within the district.

**Table 3: Numbers of interviews and observations to be conducted in facilities and at district level**

|  | **Health workers** | **Health managers** | **HFMC members** | **Observations** |
| --- | --- | --- | --- | --- |
| **District** | 0 | 2 | 0 | 0 |
| **Jibjibe PHC** | 2 (1 ANM; 1AHW) | 1 |  |  |
| **Syafrubese HP** | 2 (2 ANM) | 0 | 0 | 1 |
| **Parchang HP** | 1 (1 AHW) | 1 |  | 1 |
| **Laharepauwa HP** | 1 (1 AHW) |  | 1 | 1 |
| **Gatlan HP** | 2 (1 AHW; 1ANM) |  | 1 | 1 |
| **Total** | **8** | **4** | **2** | **4** |
